# Bone Marrow Mesenchymal Stem Cells Support Acute Myeloid Leukemia Bioenergetics and Enhance Antioxidant Defense and Escape from Chemotherapy

**DOI:** 10.1016/j.cmet.2020.09.001

**Published:** 2020-11-03

**Authors:** Dorian Forte, María García-Fernández, Abel Sánchez-Aguilera, Vaia Stavropoulou, Claire Fielding, Daniel Martín-Pérez, Juan Antonio López, Ana S.H. Costa, Laura Tronci, Efterpi Nikitopoulou, Michael Barber, Paolo Gallipoli, Ludovica Marando, Carlos López Fernández de Castillejo, Alexandar Tzankov, Sabine Dietmann, Michele Cavo, Lucia Catani, Antonio Curti, Jesús Vázquez, Christian Frezza, Brian J. Huntly, Juerg Schwaller, Simón Méndez-Ferrer

**Affiliations:** 1 Wellcome-MRC Cambridge Stem Cell Institute, CB2 0AW Cambridge, UK; 2 Department of Haematology, University of Cambridge, CB2 0AW Cambridge, UK; 3 National Health Service Blood and Transplant, CB2 0PT Cambridge, UK; 4 Centro Nacional de Investigaciones Cardiovasculares (CNIC), 28029 Madrid, Spain; 5 University Children’s Hospital and Department of Biomedicine (DBM), University of Basel, 4031 Basel, Switzerland; 6 CIBER de Enfermedades Cardiovasculares (CIBERCV), Madrid, Spain; 7 MRC Cancer Unit, University of Cambridge, CB2 0XZ Cambridge, UK; 8 Institute of Pathology, University Hospital Basel, 4031 Basel, Switzerland; 9 Istituto di Ematologia “Seràgnoli”, Dipartimento di Medicina Specialistica, Diagnostica e Sperimentale, Università degli Studi, 40138 Bologna, Italy; 10 Azienda Ospedaliero-Universitaria di Bologna, via Albertoni 15, 40138 Bologna, Italy

**Keywords:** bone marrow mesenchymal stem cells, acute myeloid leukemia, microenvironment, OXPHOS, TCA cycle, glutathione, antioxidant, chemotherapy, metabolic adaptation, hematopoietic stem cell niche

## Abstract

Like normal hematopoietic stem cells, leukemic stem cells depend on their bone marrow (BM) microenvironment for survival, but the underlying mechanisms remain largely unknown. We have studied the contribution of nestin^+^ BM mesenchymal stem cells (BMSCs) to MLL-AF9-driven acute myeloid leukemia (AML) development and chemoresistance *in vivo*. Unlike bulk stroma, nestin^+^ BMSC numbers are not reduced in AML, but their function changes to support AML cells, at the expense of non-mutated hematopoietic stem cells (HSCs). Nestin^+^ cell depletion delays leukemogenesis in primary AML mice and selectively decreases AML, but not normal, cells in chimeric mice. Nestin^+^ BMSCs support survival and chemotherapy relapse of AML through increased oxidative phosphorylation, tricarboxylic acid (TCA) cycle activity, and glutathione (GSH)-mediated antioxidant defense. Therefore, AML cells co-opt energy sources and antioxidant defense mechanisms from BMSCs to survive chemotherapy.

## Highlights

• Nestin^+^ BMSCs support leukemogenesis and chemoresistance
• BMSCs support metabolic requirements of LSCs
• BMSCs provide LSCs with essential antioxidant defense from chemotherapy
• GSH and GSH peroxidases underlie BMSC-derived antioxidant AML protection

## Graphical Abstract

**Figure:**
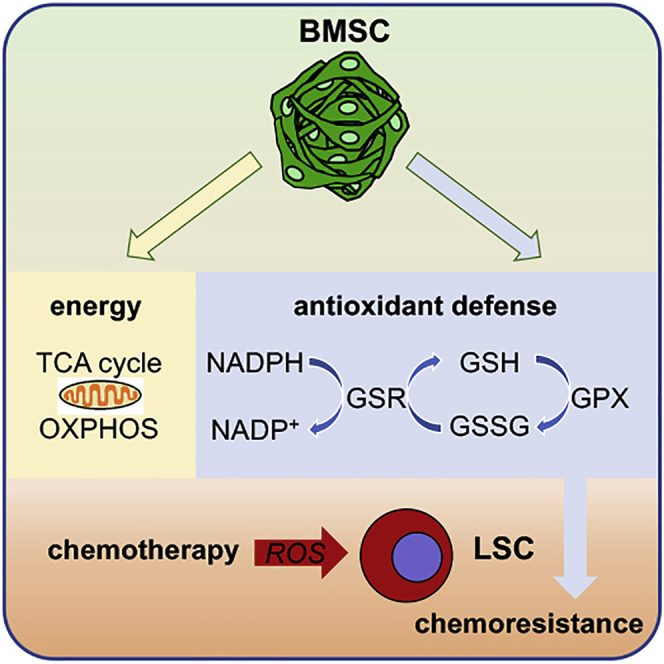

## Teaser

Forte et al. reveal that nestin+ bone marrow stromal cells directly contribute to leukemogenesis and chemotherapy resistance in an *in vivo* model of acute myeloid leukemia. Nestin+ BMSCs support leukemic stem cells through a dual mechanism of increased bioenergetic capacity through OXPHOS and TCA and glutathione-dependent antioxidant defense.

## Context and Significance

**Several lines of evidence indicate that the microenvironment plays a key role in leukemia development, chemoresistance, and, more recently, resistance to immunotherapy. Therefore, dissecting and targeting niche-driven mechanisms of resistance might provide key adjuvant therapies to eradicate leukemia. This study shows that BMSCs expressing the marker nestin directly support leukemia stem cell (LSC) survival and chemoresistance. Nestin^+^ BMSCs increase energy production in LSCs through increased TCA cycle and oxidative phosphorylation (OXPHOS) and simultaneously provide LSCs with key antioxidant tools necessary to balance ROS levels during leukemogenesis and chemotherapy. GSH-dependent antioxidant pathways emerge as key players in the BMSC-LSC crosstalk and represent potential targets for adjuvant therapies in AML.**

## Introduction

Acute myeloid leukemia (AML) is a cancer caused by accumulation of poorly differentiated hematopoietic cells, which infiltrate different tissues (Döhner et al., 2015). AML initiates through genetic or epigenetic lesions in hematopoietic stem cells (HSCs) or myeloid progenitors that can transform into LSCs, which are chemoresistant and cause tumor relapse (Bonnet and Dick, 1997; Huntly and Gilliland, 2005; Krivtsov and Armstrong, 2007; Krivtsov et al., 2006; Lapidot et al., 1994). Translocations between the *mixed lineage leukemia 1* gene (*MLL1*) and other partner genes are a molecular hallmark of pediatric and often therapy-related adult acute leukemia. Subsequently, they are associated with frequent relapse and poor prognosis with current therapies. One of the most common MLL translocations generates the MLLT3-AF9 fusion gene (Krivtsov and Armstrong, 2007; Muntean and Hess, 2012; Tan et al., 2011). Murine models of MLL-AF9 reproduce the human pathology (Corral et al., 1996; Krivtsov et al., 2006; Milne, 2017) and generate a more aggressive disease when the fusion oncogene is expressed in HSCs, compared with more mature progenitors (George et al., 2016; Krivtsov et al., 2013; Stavropoulou et al., 2016). Resembling normal HSCs, LSCs rely on their local microenvironment for survival and chemoresistance (Méndez-Ferrer et al., 2020). However, the underlying *in vivo* mechanisms remain incompletely understood.

Although LSCs share some features with normal HSCs, their metabolism is reprogrammed to meet high energy and biomass production demands in AML (Baccelli et al., 2019; Gallipoli et al., 2018). Although many cancer cells utilize aerobic glycolysis for energy production (Warburg et al., 1927), cancer stem cells or chemoresistant cells in different tumors (including AML) rely on mitochondrial oxidative phosphorylation (OXPHOS) for their high metabolic demand (Baccelli et al., 2019; Farge et al., 2017; Jacque et al., 2015; Lagadinou et al., 2013; Molina et al., 2018; Pollyea et al., 2018). However, the cellular and molecular basis underlying the metabolic reprogramming of the LSC niche is largely unknown. Mitochondrial transfer from BM mesenchymal stem cells (BMSCs) to AML cells *in vitro* has been recently described as a mechanism that provides AML cells with additional energy. This transfer increases upon chemotherapy and was proposed as an additional mechanism of resistance by reducing mitochondrial depolarization (Marlein et al., 2017; Moschoi et al., 2016). However, AML cells have abnormally high reactive oxygen species (ROS) levels (Li et al., 2011), and it remains unclear how AML cells are able to cope with the additional ROS resulting from increased mitochondrial content. Indeed, cellular proliferation and survival depend on critically fine-tuned levels of ROS, which are mainly generated by the mitochondria. AML cells are able to maintain relatively high ROS levels without reaching a cytotoxic level through increased activity of antioxidant pathways (Li et al., 2011). However, the possible role of the microenvironment in balancing ROS levels and providing AML cells with antioxidant defense *in vivo* remains largely unexplored.

BMSCs expressing the intermediate filament protein nestin provide HSC niche function (Méndez-Ferrer et al., 2010) and largely overlap with BMSCs labeled in subsequent studies using alternative markers (Ding et al., 2012; Greenbaum et al., 2013; Mende et al., 2019; Méndez-Ferrer, 2019; Omatsu et al., 2010; Park et al., 2012). Nestin^+^ niches are reduced in humans or mice with chronic myeloproliferative neoplasms (Arranz et al., 2014), which can be considered preleukemic disorders due to their higher incidence of leukemic transformation. In contrast, increased number of BM nestin^+^ cells have been reported in AML mice transplanted with serially passaged hematopoietic cells transformed with a retrovirally expressed *MLL-AF9* fusion oncogene (Hanoun et al., 2014). However, whether and how nestin^+^ cells play a role during leukemogenesis remain unknown.

Here, we have studied the contribution of nestin^+^ cells to MLL-AF9-driven AML development and resistance to conventional chemotherapy *in vivo*. The results demonstrate that nestin^+^ cells contribute to AML progression by increasing the bioenergetic capacity of AML cells and facilitating their chemoresistance. BMSC-dependent increased OXPHOS, tricarboxylic acid (TCA) cycle and glutathione (GSH)-mediated antioxidant defense against excessive ROS allow AML cells to meet their high metabolic demands and survive chemotherapy. Targeting these BMSC-dependent AML survival pathways can synergize with conventional chemotherapy to eliminate chemoresistant cells.

## Results

### Unlike Bulk Stromal Cells, Nestin^+^ Niche Cells Are Preserved in AML

**Figure 1:**
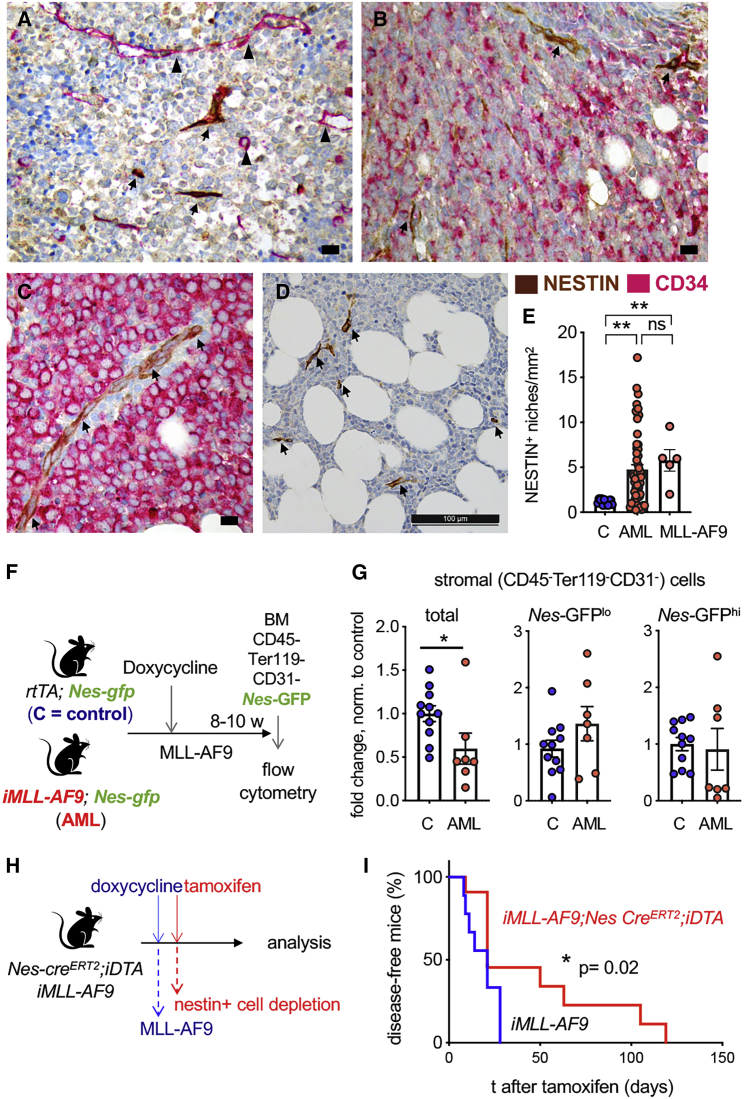
Unlike Bulk BM Stromal Cells, Nestin+ Niche Cells Are Preserved in Human and Murine AML and Promote Leukemogenesis (A–D) Representative examples of immunohistochemistry for human NESTIN (brown) and human CD34 (pink) in BM samples from AML patients. CD34− AML (A), CD34+ AML ([B] and [C]), and MLL-AF9+ AML (D) show increased NESTIN+ niches (arrows) and CD34+ vessels (arrowheads). Scale bar, 10 μm (A–C), 100 μm (D). (E) Quantification of NESTIN+ niches from (A and B) (control n = 12; MLL-AF9− AML n = 56; MLL-AF9+ AML n = 5). ^∗^p < 0.05, ^∗∗^p < 0.01, one-way ANOVA and Bonferroni comparisons. (F) Scheme showing the induction of AML in primary, non-transplanted *Nes*-GFP mice to study BMSC changes during leukemogenesis. (G) Fold change in the number of BM stromal cells (CD45− Ter119−CD31−) and BMSCs expressing low or high levels of *Nes*-GFP (NesGFPlow/high) in the BM of control (C) *Nes*-GFP;*rtTA* mice and *Nes*-GFP;*rtTA;iMLL-AF9* (AML) mice. Numbers were normalized with the average of WT controls in each independent experiment. Mice were analyzed 8–10 weeks after inducing MLL-AF9 expression. Dots represent data from individual mice (n = 2 independent experiments). Data are mean ± SEM. Unpaired two-tailed t test. (H) Scheme showing experimental depletion of nestin+ cells in primary, non-transplanted leukemic mice. MLL-AF9 expression *(iMLL-AF9)* is induced with doxycycline. (I) Nestin+ cell depletion extends AML mouse survival. Kaplan-Meier survival curve of primary iMLL-AF9 mice in control group (black, n = 9) or after nestin+ cell depletion (red, n = 11). Logrank test.

Nestin^+^ BMSCs are HSC niche-forming cells (Méndez-Ferrer et al., 2010), which have been reported to undergo diametrically opposite numerical changes in different myeloproliferative disorders (Arranz et al., 2014; Hanoun et al., 2014). To investigate whether nestin^+^ BM cells are affected in human AML, we performed immunohistochemistry for the human NESTIN protein in BM biopsies from MLL-AF9^+^ (n = 5) or MLL-AF9^−^ (n = 56) AML patients and control donors (n = 12) (Figures 1A–1C). BM NESTIN^+^ niches increased 4–5-fold in AML patients regardless of the presence of the MLL-AF9 translocation (Figures 1D and 1E). These results are consistent with findings in a retrovirally induced MLL-AF9 AML mouse model (Hanoun et al., 2014) and contrast with reduced NESTIN^+^ niches in human or mouse MPN (Arranz et al., 2014; Drexler et al., 2019).

To reproduce these findings in an inducible AML mouse model, we have used the doxycycline-inducible rtTA;MLL-AF9 (referred as iMLL-AF9) mouse strain (Stavropoulou et al., 2016). Both primary AML mice following induction and irradiated C57BL/6 recipient mice transplanted with iMLL-AF9 BM cells develop myelo-monoblastic AML that closely mimics the human disease (Stavropoulou et al., 2016). To visualize nestin^+^ cells, iMLL-AF9 mice were intercrossed with *Nestin*-GFP reporter mice, which express the green fluorescent protein (GFP) under the regulatory elements of the nestin (*Nes*) promoter (Méndez-Ferrer et al., 2010; Mignone et al., 2004). AML was induced *in vivo* with doxycycline (Figure 1F). Nestin-GFP^high^ cells are associated with central arterioles and also with transition zone vessels that connect arterioles with sinusoids near the bone (García-García et al., 2019; Itkin et al., 2016; Kunisaki et al., 2013; Kusumbe et al., 2014). Nestin-GFP^low^ cells coincide with LepR^+^ cells in central BM sinusoids (Ding et al., 2012). *Nes*-GFP*;iMLL-AF9* mice sacrificed 8–10 weeks after AML induction showed a significant reduction in the number of BM CD45^−^Ter119^−^CD31^−^ stromal cells, compared with disease-free controls (Figure 1G). However, we observed highly variable but overall unchanged numbers of Nestin-GFP^high/low^ BMSCs. The observation of selective retention of nestin^+^ cells raised our interest to study whether these cells might play an active role in leukemogenesis.

### Nestin^+^ Cells Promote AML Development

The persistence of nestin^+^ cells in highly infiltrated BM of AML patients and mice suggested that these cells may actively contribute to disease progression, possibly by providing support to leukemic blasts, as previously suggested for other stromal cells, albeit through *in vitro* rather than *in vivo* studies (Brenner et al., 2017; Corradi et al., 2018; Geyh et al., 2016; Kornblau et al., 2018; Wu et al., 2018; Yehudai-Resheff et al., 2019). To assess the role of nestin^+^ cells *in vivo*, we took advantage of a mouse model that allows conditional depletion of nestin^+^ cells. We intercrossed mice carrying tamoxifen-inducible Cre^ERT2^ recombinase under the control of *Nes* regulatory elements (*Nes-Cre*^*ERT2*^) (Balordi and Fishell, 2007) with the *R26lacZbpA*^*flow*^*DTA* strain (Brockschnieder et al., 2006), which harbors a Cre-inducible diphtheria toxin A allele allowing for conditional depletion of nestin^+^ cells upon tamoxifen treatment (Figure S1A). As early as 25 days after tamoxifen application, *Nes-cre*^*ERT2*^*;R26lacZbpA*^*flox*^*DTA* mice (abbreviated as *Nes-cre*^*ERT2*^;*iDTA*) showed decreased numbers of BMSCs measured functionally (by colony-forming unit fibroblasts, CFU-F) or immunophenotypically (CD90^+^), whereas CD31^+^ BM endothelial cells appeared unaffected (Figures S1B–S1D). Conditional depletion of nestin^+^ BM cells in healthy mice was associated with 2-fold decreased HSC activity, measured by long-term competitive repopulation assays (Figure S1E). These results confirm the HSC niche dependence on nestin^+^ BMSCs (Méndez-Ferrer et al., 2010) and validate the nestin^+^ cell depletion model.

To test the role of nestin^+^ cells in AML, Nes*-cre*^*ERT2*^;*iDTA* mice were intercrossed with *iMLL-AF9* mice. Compound *iMLL-AF9; Nes-cre*^*ERT2*^;*iDTA* mice and their control littermates were administered doxycycline to induce AML and tamoxifen to deplete nestin^+^ cells (Figure 1H). Notably, the elimination of nestin^+^ cells upon AML development significantly extended mouse survival (Figure 1I), suggesting that nestin^+^ cells promote leukemogenesis *in vivo*.

### AML Cells Hijack Nestin^+^ Niche Cells to Promote Leukemogenesis

**Figure 2:**
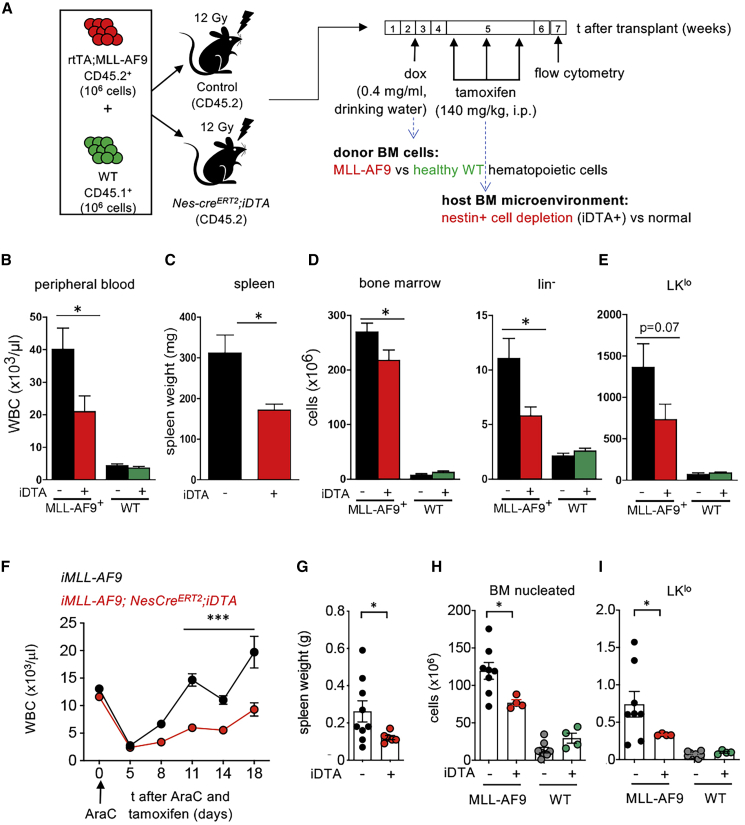
Nestin+ Cells Promote Leukemia Chemoresistance In Vivo (A) Scheme showing the experimental setting to simultaneously study the impact of nestin+ cell depletion on healthy and leukemic hematopoietic cells. Lethally irradiated CD45.2 control mice or *Nes-cre^ERT2^*;iDTA mice were transplanted with 106 iAML *(rtTA;MLL-AF9)* CD45.2+ BM nucleated cells and 106 CD45.1+ WT BM nucleated cells. Doxycycline administration started 2 weeks after transplant; tamoxifen was administered 4 weeks post-transplant and mice were sacrificed and analyzed 4 weeks later. (B–D) Number of WT and MLL-AF9+ WBC (B), spleen weight (C), BM nucleated, and lineage-negative cells (D). (E) BM lin−ckitlosca1− (LKlo) cells. Data in (D and E) represent the cellularity of 4 limbs, sternum, and spine (n = 18 mice/group, pooled from 3 independent experiments). (F–I) Nestin+ cells support chemoresistance in AML mice. (F) WBCs, (G) spleen weight, (H) BM nucleated cells, and (I) LKlo cells in control (iDTA−) or *Nes-creERT2;iDTA* (iDTA+) mice transplanted with a mixture of WT and iMLL-AF9 BM cells, receiving tamoxifen and AraC treatment simultaneously, as in Figure S1A (n = 4–8). Dots represent data from individual mice. Data are mean ± SEM. ^∗^p < 0.05; ^∗∗∗^p < 0.001; unpaired two-tailed t test.

The results thus far suggested that BM nestin^+^ cells not only support normal HSCs in wild type (WT) (healthy) mice (Méndez-Ferrer et al., 2010) (Figure S1E) but also support leukemogenesis in mice (Figure 1H). To directly compare the role of nestin^+^ cells in normal hematopoiesis and leukemia development, *Nes-cre*^*ERT2*^;*iDTA* and their littermate *iDTA* controls were competitively transplanted with WT BM cells and non-induced iMLL-AF9 BM cells, allowing simultaneous monitoring of normal and leukemic hematopoiesis, which were distinguished in the same animal by the expression of different CD45 surface marker isoforms (Figure 2A). We observed that depletion of nestin^+^ cells during the course of AML development selectively diminished the number of MLL-AF9^+^ cells in peripheral blood, spleen, and BM and reduced the number of leukemic hematopoietic-lineage-negative (lin^−^) progenitor cells, without affecting normal hematopoiesis (Figures 2B–2D). Among immature hematopoietic cells, lin^−^ckit^low^sca1^−^ cells (LK^lo^) cells preferentially expanded by the MLL-AF9 oncogene and tended to decrease upon nestin^+^ cell depletion (Figures 2E, S1F, and S1G). This result suggests that AML cells instruct nestin^+^ cells to promote leukemogenesis at the expense of their role supporting normal HSCs.

### Nestin^+^ Cells Support *In Vivo* Chemoresistance in AML

Since nestin^+^ cells support AML cells, we next tested their potential to maintain resistant cells and help them evade AML chemotherapy. For that purpose, nestin^+^ cell depletion was combined with cytarabine (AraC—a chemotherapeutic drug frequently used in AML therapy) (Burnett et al., 2011; Tamamyan et al., 2017) in mice transplanted simultaneously with WT and iMLL-AF9 cells (Figure S1H). Independently of nestin^+^ cell depletion, AraC significantly reduced the peripheral white blood cell (WBC) counts. However, compared with control mice, AML relapse was significantly delayed in *Nes-cre*^*ERT2*^*;iDTA* mice (Figure 2F). As a result, splenomegaly was only detected at the time of analysis in symptomatic control mice treated with chemotherapy only, but not in those with nestin^+^ cell depletion (Figure 2G). Consistent with the previous results, the combination of AraC with nestin^+^ cell depletion significantly reduced leukemic cells but did not affect non-leukemic BM hematopoietic cells (compare Figures 2D and 2H). Combined AraC and nestin^+^ cell depletion preferentially decreased LK^lo^ cells (Figure 2I), whereas it did not alter leukemic/non-leukemic LSK/LK cells (Figures S1I and S1J). Altogether, these results suggest that AML cells highjack HSC niche functions of nestin^+^ cells to evade chemotherapy.

### BMSCs Support Leukemic Blast Survival and Chemoresistance *In Vitro*

**Figure 3:**
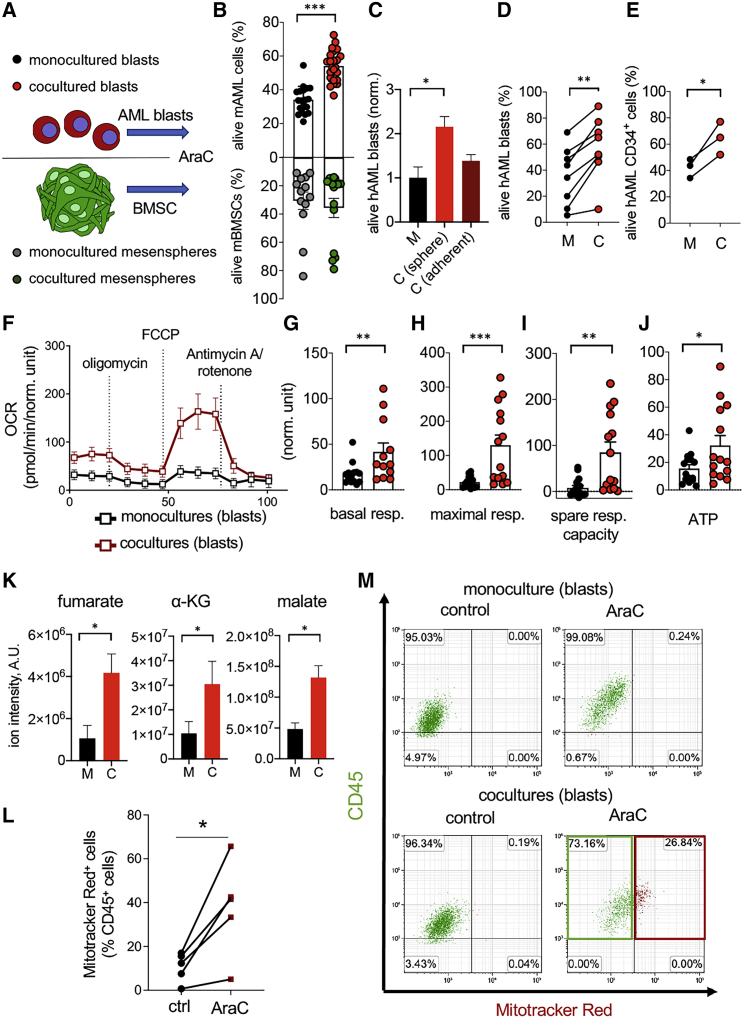
BMSCs Support Leukemic Blast Survival, Chemoresistance, and Bioenergetics (A) Scheme showing the different culture conditions and cells tested. (B) Frequency of alive (AnnexinV−DAPI−) cells 24 h after AraC treatment (n = 19). (C) Frequency of alive human AML cells monocultured (M) or cocultured or cocultured (C) with human mesenspheres or adherent BMSCs for 24 h under AraC treatment (n = 3–5). ^∗^p < 0.05; One-way ANOVA and Bonferroni comparisons. (D and E) Frequency of alive CD45+ (D, n = 8) cells or CD34+ HSPCs (E, n = 3) from human AML BM 24 h after AraC in monoculture (black) or coculture (red) with BMSCs expanded as mesenspheres from AML patients (n = 8). ^∗^p < 0.05; ^∗∗^p < 0.01; paired two-tailed t test. (F) OCR indicating mitochondrial respiration after oligomycin, FCCP and antimycin A/rotenone treatment in leukemic blasts cultured alone or previously cocultured with mesenspheres for 24 h in presence of AraC. (G–J) Seahorse measurement of basal respiration (G), maximal respiration (H), spare respiratory capacity (I), and ATP content (J) in murine leukemic blasts monocultured (black columns) or cocultured with BMSCs (red columns); n = 6 independent experiments. Data represent mean ± SEM. ^∗^p < 0.05; ^∗∗^p < 0.01; ^∗∗∗^p < 0.001; unpaired two-tailed t test. (K) Levels of the TCA cycle metabolites fumarate, ɑ-KG, and malate measured by LC-MS in murine leukemic blasts monocultured (black columns) or cocultured with BMSCs (red columns); n = 5 independent experiments with 4 biological replicates each. (L) Frequency of AML blasts uptaking MitoTracker Red+ mitochondria previously stained in BMSCs only, before the coculture with AML cells under AraC treatment; n = 4 independent experiments; ^∗^p < 0.05; paired two-tailed t test. (M) Representative flow cytometry diagrams showing CD45 (green) and MitoTracker Red (red) fluorescence in murine AML blasts monocultured or cocultured with BMSCs, which were previously stained with MitoTracker Red to label their mitochondria. The frequencies of gated cell populations are indicated. The red box highlights the increased frequency of BMSC-derived mitochondria in AML cells after AraC treatment.

To investigate the mechanisms by which nestin^+^ cells support AML development and chemoresistance, we optimized an *in vitro* system to grow human or murine NESTIN^+^ BMSCs as mesenspheres, which exhibit increased *in vivo* self-renewal and support of HSCs, compared with standard plastic-adherent BMSCs (Ghazanfari et al., 2016; Isern et al., 2013; Méndez-Ferrer et al., 2010). Upon coculturing mesenspheres with AML blasts (Figure 3A), more leukemic blasts survived AraC treatment (Figure 3B). Importantly, human mesenspheres provided enhanced chemoprotection of human AML blasts, compared with plastic-adherent BMSCs from the same donors (Figure 3C). Chemoprotection was similarly detected upon coculturing human mesenspheres with human BM CD45^+^ blasts or human CD34^+^ HSC-enriched cells (Figures 3D and 3E). These data suggest that nestin^+^ BMSCs support survival and chemotherapy escape of mouse and human AML cells *in vitro*.

### BMSCs Enhance Leukemic Blast Bioenergetics by Increasing OXPHOS and TCA Cycle

Cumulative evidence suggest that the BM microenvironment provides critical metabolic support to AML cells (Méndez-Ferrer et al., 2020). Therefore, metabolic studies were conducted to investigate the mechanisms explaining increased AML survival in coculture with BMSCs. Although most cancer cells fulfill their metabolic requirements by aerobic glycolysis (Warburg et al., 1927), cancer stem cells or chemoresistant cells in different tumors, including AML, seem to rely on mitochondrial OXPHOS (Baccelli et al., 2019; Farge et al., 2017; Jacque et al., 2015; Molina et al., 2018; Pollyea et al., 2018). To investigate the possible metabolic dependency of leukemic blasts from BMSCs, we measured the oxygen consumption rate (OCR)—a readout of mitochondrial function—in leukemic cells previously cultured for 24 h alone or in coculture with BM mesenspheres in presence of AraC. OCR significantly increased in leukemic blasts, which had been previously cocultured with BM mesenspheres, compared with blasts cultured alone (Figure 3F). The basal and maximal respiratory rate of AraC-treated leukemic blasts increased >5-fold in the presence of BMSCs (Figures 3G and 3H). The spare respiratory capacity, which reflects the difference between the maximal and the basal respiratory rate, increased 10-fold in coculture (Figure 3I). Consequently, ATP production was doubled in coculture, compared with monoculture (Figure 3J). Together, these results suggest that BMSCs promote AML cell survival under chemotherapy by increasing their bioenergetic capacity.

To further characterize the metabolic profile of chemotherapy-treated leukemic blasts, we analyzed the intracellular metabolite levels by liquid chromatography-mass spectrometry (LC-MS). In coculture with mesenspheres, leukemic blasts exhibited 2–3-fold higher levels of the TCA cycle intermediates fumarate, α-ketoglutarate (α-KG), and malate (Figure 3K), suggesting that BMSCs stimulate TCA cycle in AML cells. Since TCA cycle takes place in the mitochondria, we labeled the mitochondria of BMSCs and AML cells using different fluorochromes. After AraC treatment in coculture, the transfer of mitochondria from BMSCs to leukemic blasts was observed as a potential source of increased energy (Figures 3L and 3M), confirming and extending previous observations with plastic-adherent cells (Marlein et al., 2017; Moschoi et al., 2016).

### BMSCs Increase Antioxidant Defenses in Leukemic Blasts Facilitating Chemoresistance

**Figure 4:**
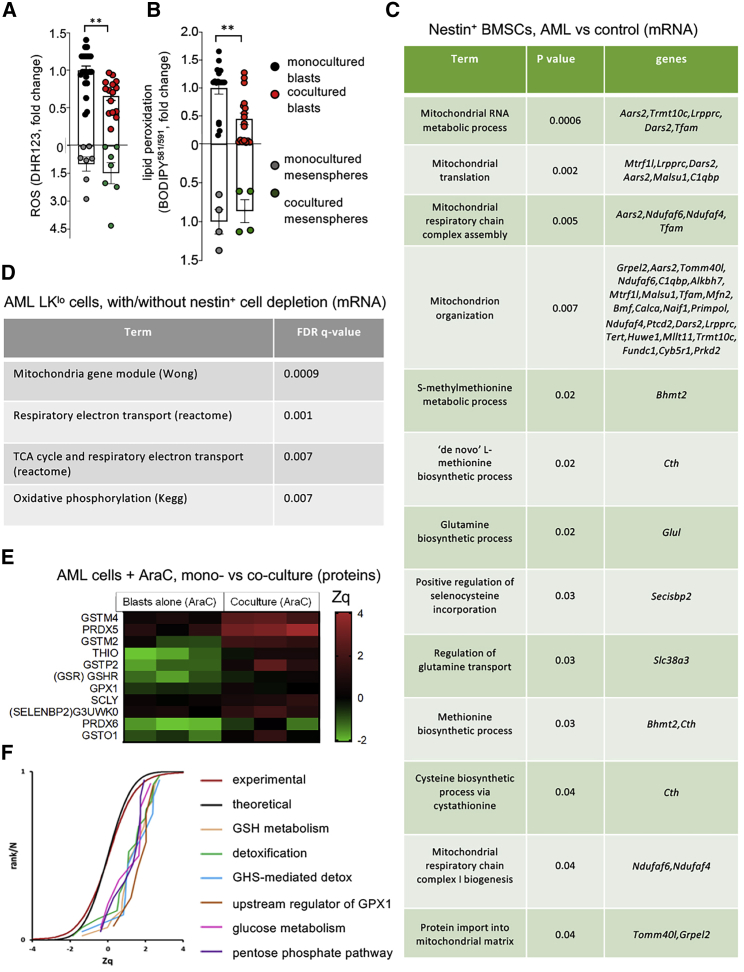
BMSCs Provide Leukemic Blasts with Antioxidant Defense during Chemotherapy (A and B) ROS measured by DH123 staining (A) and lipid peroxidation detected with BODIPY^581/591^ (B) in AML blasts and BMSCs cultured alone or together for 24 h in presence of AraC. Each dot is a biological replicate. ^∗∗^p < 0.05; unpaired two-tailed t test. (C) Selected pathways from the GO enrichment analysis of significantly upregulated mRNA (RNA-seq) of CD45−CD31−Ter119−Nes-GFP+ cells isolated from the BM of primary iMLL-AF9 mice or control mice. The GO term, adjusted p value and differentially expressed genes in each category are indicated. (D) Selected pathways from the GSEA analysis of RNA-seq of hematopoietic-lineage-negative ckitlow (LKlo) cells isolated from the BM of AML mice with or without nestin+ cell depletion. Lethally irradiated CD45.2 control mice or *Nes-creERT2;iDTA* mice were transplanted with 106 iAML *(rtTA;MLL-AF9)* CD45.2+ BM nucleated cells and 10^6^ CD45.1+ WT BM nucleated cells. Doxycycline administration started 2 weeks after transplant; tamoxifen was administered 4 weeks post-transplant; and mice were sacrificed and analyzed 4 weeks later. BM MLL-AF9+ lin− ckit^low^ (LK^lo^) cells were sorted from leukemic mice with or without nestin+ cell depletion. The pathway term, signature, and adjusted p value are indicated. (E) Heatmap showing antioxidant and GSH related among the top upregulated (red) protein pathways detected by proteomics in AraC-treated AML blasts cultured alone or cocultured with mesenspheres for 24 h. Quantitative proteomics results were analyzed using the WSPP statistical model (Navarro et al., 2014) and expressed in log2 fold change values in units of SD (Zq) with respect to baseline. Significant protein changes (adjusted p < 0.05) are indicated; two-sided Fisher test, 3 biological replicates in each condition. (F) SBT analysis (García-Marqués et al., 2016) to detect changes in functional categories due to coordinated protein behavior. The analysis detected significantly increased pathways (positive values of Zq indicate increased protein changes) compared with the theoretical N(0,1) distribution (black curve); for comparison, the distribution of Zq values of all the proteome is also shown (red curve). The panel displays the cumulative distributions of Zq from proteins belonging to a set of representative altered categories, showing the high coordination of protein responses in these pathways.

The mitochondrial transfer from BMSCs to AML cells raised the question of its possible impact on cellular ROS. Indeed, AML cells already exhibit abnormally high mitochondrial-derived ROS at baseline (Li et al., 2011), and it is unclear how AML cells can survive putatively additional ROS derived from BMSC-derived mitochondria (Figures 3L and 3M). To address this question, we labeled mitochondrial ROS with the fluorescent dye DHR123. As expected (Amarante-Mendes et al., 1998), AraC treatment significantly increased ROS levels in the leukemic blasts (Figure S2A). Interestingly, despite mitochondrial uptake and increased OXPHOS, AML cells cocultured with BMSCs showed decreased ROS levels (Figure 4A). Excessive ROS levels can cause cell damage and death by inducing lipid peroxidation (Barrera, 2012). Like ROS, lipid peroxidation similarly decreased in leukemic blasts cocultured with mesenspheres (Figure 4B), indicating that BMSCs protect AML cells from chemotherapy-derived ROS-induced cell damage. These effects required cell contact between BMSCs and AML cells since they were not reproduced in transwell coculture or using conditioned medium (Figures S2B–S2E).

### The Glutathione System Mediates Antioxidant Protection from Chemotherapy by BMSCs

To investigate potential microenvironmental pathways increasing the ability of AML cells to buffer ROS, we performed RNA-seq in CD45^−^CD31^−^Ter119^−^*Nes*-GFP^+^ BMSCs isolated from control WT mice or primary AML mice upon leukemia development. Whereas ~900 genes were upregulated, ~600 genes appeared downregulated in nestin^+^ BMSCs from AML mice (Table S1). GO enrichment analysis of significantly upregulated genes in AML mice showed 54 genes belonging to the mitochondrion and highlighted mitochondrial-associated pathways, such as mitochondrial translation, mitochondrion organization, and mitochondrial respiratory chain complex assembly and biogenesis (Figure 4C; complete analysis is shown in Table S1). Interestingly, several genes belonging to antioxidant pathways were increased, including selenocysteine regulation of methionine biosynthesis, which is essential for the generation of the cellular antioxidant GSH. To investigate the corresponding transcript alterations in the AML cells regulated by nestin^+^ BMSCs, we performed RNA-seq in BM LK^lo^ cells obtained from AML mice with/without nestin^+^ cell depletion. Consistent with the transcriptomics changes observed in BMSCs, gene set enrichment analysis (GSEA) showed significantly increased mitochondrial pathways, such as OXPHOS and TCA cycle, in AML LK^lo^ cells following nestin^+^ cell depletion (Figures 4D and S2F; Table S2).

**Figure 5:**
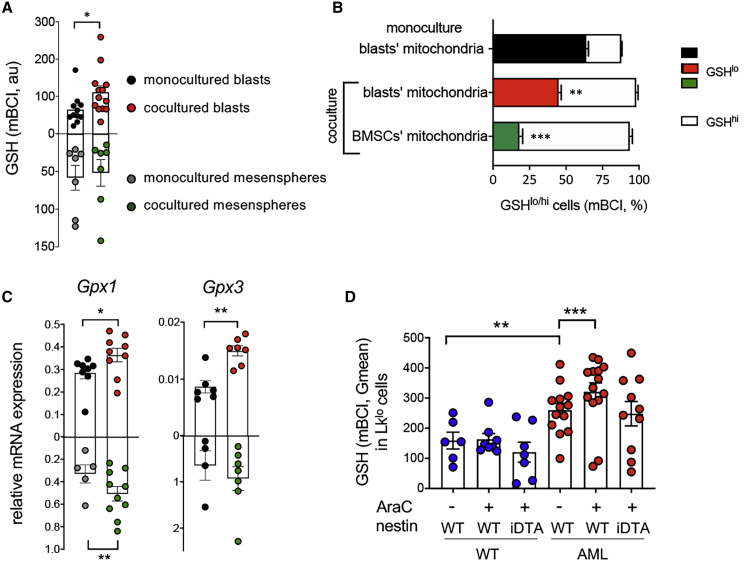
BMSC-Dependent GSH-Related Pathways Protect AML Cells from Chemotherapy (A) Reduced glutathione (GSH) measured by mBCI staining in AML blasts and BMSCs cultured alone or together for 24 h in presence of AraC. (B) Frequency of AraC-treated GSH^lo/hi^ AML cells monocultured (upper bar) or cocultured with BMSCs (middle and lower bar), separating leukemic blasts, which contain mitochondria derived from previously labeled BMSCs (lower bar) from those containing only blasts’ mitochondria (middle and upper bars); n = 4–5. (C) mRNA expression of *Gpx1* and *Gpx3* in AML blasts and BMSCs cultured alone or together for 24 h in presence of AraC. (D) GSH measured by mBCI staining in LK^lo^ WT or *iMLL-AF9* cells from mice with/without nestin+ cell depletion (*Nes-cre^ERT2^;iDTA* and control littermates) treated as in Figure S3H. Mice were treated with standard chemotherapy (AraC+) or vehicle (AraC−). (A, C, and D) Each dot represents a biological replicate. Data represent mean ± SEM. ^∗^p < 0.05; ^∗∗^p < 0.01; ^∗∗∗^p < 0.001. Unpaired two-tailed t test. (B and D) One-way ANOVA followed by pairwise Bonferroni comparisons.

To further investigate the functional consequences of these transcriptomics alterations in nestin^+^ BMSCs and, particularly, when leukemic cells are challenged by chemotherapy, we performed proteomic analysis of AraC-treated AML blasts monocultured or cocultured with mesenspheres for 24 h. These analyses revealed differential abundance of proteins (Table S3), including 11 antioxidant proteins associated with detoxification and/or GSH metabolism, which were significantly upregulated in coculture (Figure 4E). The quantified proteins were grouped into ontological categories from GO, KEGG, or REACTOME databases, and the systems biology triangle (SBT) model (García-Marqués et al., 2016) was used to analyze coordinated increase or decrease of proteins belonging to these categories. The analysis revealed statistically significant coordinated increases in the proteins belonging to glucose metabolism, pentose phosphate pathway (PPP), GSH metabolism and detoxification, including upstream regulators of GSH peroxidase 1 (Gpx1), which oxidizes GSH to GSSG for detoxification (Figure 4F). Therefore, we measured reduced GSH in AraC-treated leukemic blasts and found it significantly increased in coculture with mesenspheres, compared with monoculture (Figure 5A). To correlate GSH levels with mitochondrial transfer, the mitochondria of BMSCs and leukemic blasts were stained with different colors before coculture. Remarkably, GSH levels were highest in those leukemic blasts that had uptaken mitochondria from BMSCs (Figure 5B). Increased GSH also correlated with augmented mRNA expression of key detoxifying enzymes that utilize GSH as a substrate. Notably, mRNA expression of *Gpx1* and *Gpx3* increased in the cocultured leukemic blasts (Figure 5C), confirming the proteomic results, whereas this was not the case for other relevant detoxifying enzymes (Figures S3A–S3G). These results suggest that BMSCs specifically protect leukemic cells from chemotherapy-induced ROS by increasing GSH availability and utilization, mainly via the Gpx system.

To address *in vivo* the relevance of the GSH-dependent protection of AML cells from excessive ROS associated with chemotherapy by BMSCs, *Nes-cre*^*ERT2*^;*iDTA* mice and their littermate controls were competitively transplanted with normal and iMLL-AF9 BM cells. Doxycycline was administered after 2 weeks to induce AML, and nestin^+^ cells were depleted by tamoxifen administration upon leukemia development, i.e., once peripheric blood showed high WBC (see STAR Methods), around 5 weeks later (Figure S3H). Mice were acutely injected after 24 h with a single dose of AraC and 24 h later BM LK^lo^ cells were analyzed, as this cell population preferentially expanded in the leukemic mice dependently on nestin^+^ cells (Figure 2E). GSH levels were significantly higher in leukemic LK^lo^ cells than in WT LK^lo^ cells. Moreover, GSH levels increased in leukemic LK^lo^ (but not in WT cells) 24 h after AraC application. Despite the limitations and inherent variability of this *in vivo* approach, nestin^+^ cell depletion overall impaired the capacity of AML LK^lo^ cells to boost GSH levels after chemotherapy (Figure 5D). Collectively, these results suggest that AML cells rely on BMSCs to increase their GSH-dependent antioxidant defense to escape chemotherapy.

### BMSCs Increase GSH Availability and Gpx Activity in AML Cells

**Figure 6:**
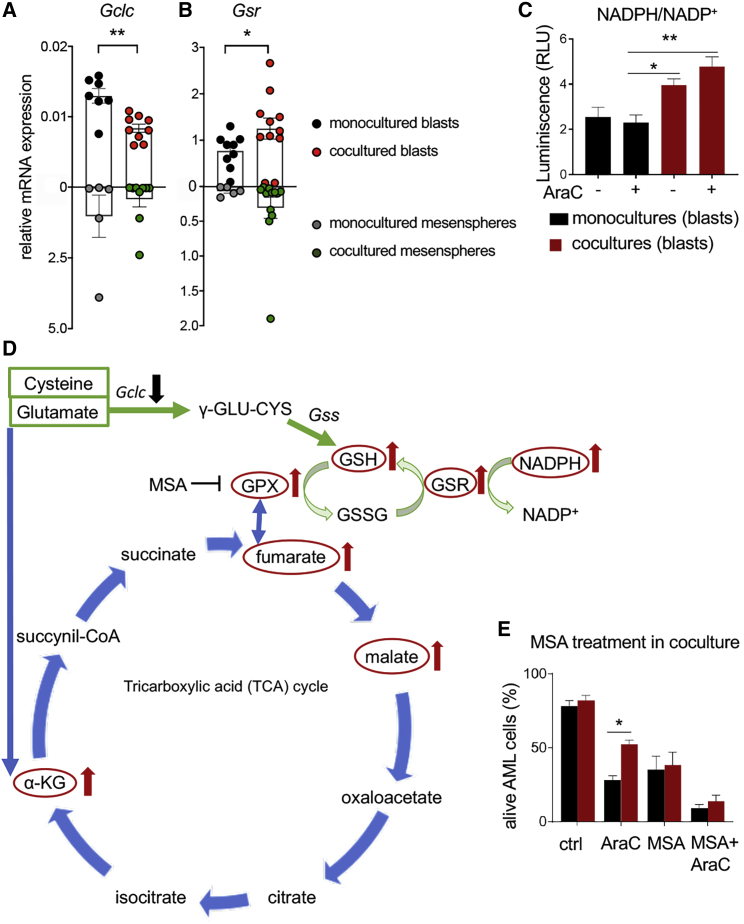
BMSCs Protect Leukemic Blasts from Chemotherapy through GSH Recycling and Oxidation by Gpx (A and B) mRNA expression of the genes encoding (A) the catalytic subunit of gamma-glutamylcysteine synthetase *(Gclc)*, which is the first rate-limiting enzyme of GSH synthesis, and (B) GSH reductase *(Gsr)*, required for GSH recycling in AML blasts and BMSCs, cultured alone (black, blasts; gray, BMSCs) or together (red, blasts; green, BMSCs) for 24 h in presence of AraC. Each dot is a biological replicate. (C) NADPH/NADP^+^ ratio in sorted CD45^+^ leukemic blasts after monoculture (black) or coculture with BMSCs (red), in the presence/absence of AraC (n = 3). (D) Schematic representation of the TCA cycle and glutathione redox cycle, indicating with arrows upregulated (red) and downregulated (black) molecules in coculture. (E) Frequency of alive (AnnexinV−DAPI−) AML cells 24 h after treatment of monoculture (black) or cocultured (red) cells with vehicle (ctrl), AraC, the Gpx inhibitor mercaptosuccinic acid (MSA, 1.6 mM) or both drugs (n = 6). (B–E) Data represent mean ± SEM. ^∗^p < 0.05; ^∗∗^p < 0.01; ^∗∗∗^p < 0.001. (B and C) Unpaired 2-tailed t test. (D–F) One-way ANOVA followed by pairwise Bonferroni comparisons.

To further study how BMSCs influence the usage of GSH by AML cells, we measured the expression of enzymes required for GSH synthesis and recycling in AML blasts. Intriguingly, mRNA expression of the catalytic subunit of gamma-glutamylcysteine synthetase (*Gclc*), which is the first rate-limiting enzyme of GSH synthesis, was reduced in AraC-treated AML blasts cocultured with BMSCs (Figure 6A), arguing against *de novo* GSH synthesis stimulated by BMSCs. However, GSH recycling following transcriptional upregulation of GSH reductase (encoded by *Gsr*) is reportedly critical for protection against ROS-induced cell death (Harvey et al., 2009). Indeed, we observed increased *Gsr* mRNA expression in AraC-treated AML cells cocultured with BMSCs (Figure 6B), validating the proteomic data (Figure 4E). Gsr requires NADPH to reduce oxidized glutathione (GSSG) and generate GSH. Supporting BMSC-mediated GSH recycling, the ratio of NADPH/NADP^+^ doubled in coculture (Figure 6C). Altogether, these metabolic studies suggest that BMSCs stimulate the TCA cycle and GSH recycling in leukemic blasts.

### Targeting BMSCs-Induced Antioxidant Defense Improves Antileukemic Chemotherapy *In Vitro* and *In Vivo*

Since the metabolomic, proteomic, and transcriptomic analyses suggested the possible relevance of Gpx1 in BMSC-dependent AML protection from chemotherapy, the Gpx1 inhibitor mercaptosuccinic acid (MSA) was tested in culture. MSA was not toxic for BMSCs at the concentration used to treat leukemic blasts (Figure S4A) and was sufficient to abrogate the chemoprotection of AML cells by the mesenspheres (Figures 6D and 6E).

**Figure 7:**
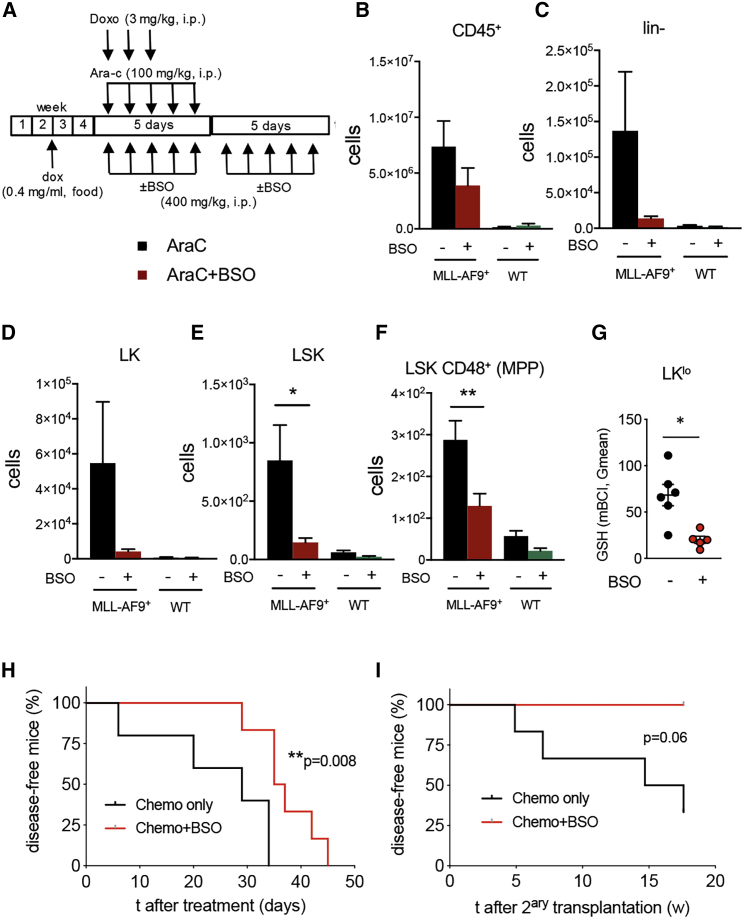
GSH Depletion Synergizes with Conventional Chemotherapy to Reduce AML Cells In Vivo (A) Experimental outline combination therapy in chimeric mice generated as depicted in Figure 3A. Lethally irradiated WT recipients were transplanted with 10^6^ iMLL-AF9;CD45.2^+^ BM cells and 10^6^ WT CD45.1^+^ BM cells. After two weeks, leukemia was induced by doxycycline in the food. Upon AML development, mice were treated with AraC-doxorubicin (Chemo) and BSO or vehicle for 10 days before flow cytometry analysis. (B–F) BM leukemic (MLL-AF9^+^) or WT (B) CD45^+^ cells, (C) hematopoietic-lineage-negative (lin^−^) cells, (D) lin^−^ckit^+^sca1^−^ (LK) cells, (E) lin^−^ckit^+^sca1^+^ (LSK) cells, and (F) LSK CD48^+^ multipotent progenitors (MPP). (G) GSH measured by mBCI staining in lin-ckit^low^ (LK^lo^) AML cells from mice treated with combined BSO therapy (red) or with standard induction chemotherapy only (black). Each dot represents a mouse. (B–G) Data are mean ± SEM. ^∗^p < 0.05; ^∗∗^p < 0.01; unpaired two-tailed t test. (H) Disease-free AML mice treated with combined BSO therapy (red) or with standard induction chemotherapy only (black; n = 6). (I) Disease-free mice after lethal irradiation and transplantation of BM cells from mice in (H) (n = 4–6). Recipient mice were fed with doxycycline-containing pellets. (H and I) Logrank test.

To test whether targeting GSH activity in AML could be therapeutic *in vivo*, the effect of GSH was studied in combination with standard chemotherapy in mice. Due to *in vivo* toxicity of MSA (Dhall et al., 2014), buthionine sulfoximine (BSO), which is a potent inhibitor of γ-glutamylcysteine synthetase (γ-GCS), was used to inhibit GSH biosynthesis *in vivo* (Tagde et al., 2014). Lethally irradiated mice were competitively transplanted with WT and non-induced iMLL-AF9 BM cells, and doxycycline was administered to induce AML. Mice were treated with chemotherapy 5 weeks after transplantation following a regimen mimicking clinical AML induction therapy. This treatment consisted of “5 + 3” i.p. injections of cytarabine (100 mg/kg/day over 5 days) and doxorubicin (3 mg/kg/day over 3 days) (Zuber et al., 2009). Mice were additionally treated with BSO (400 mg/kg/day for 10 days during and immediately after standard chemotherapy) or vehicle (Figure 7A). Notably, the combination therapy reduced 5-fold leukemic LSK cells and 2-fold leukemic LSK CD48^+^ multipotent progenitor (MPP)-like cells (but not their WT counterparts) in mice sacrificed at the same time point (see STAR Methods); similar trends were observed for Lin- and LK leukemic cells (Figures 7B–7F). Increased leukemic cell elimination was associated with 5-fold decreased GSH content in LK^lo^ putative chemoresistant leukemic cells (Figure 7G). The synergistic effect of BSO and standard chemotherapy significantly extended mouse survival (Figure 7H). Transplantations of BM cells from these mice into lethally irradiated recipients showed a marked reduction of LSC activity after combination therapy (Figure 7I). Collectively, these findings indicate that neighboring BMSC can provide AML cells with increased GSH-Gpx activity to evade chemotherapy and that this chemoprotection mechanism can be therapeutically targeted to improve outcomes.

## Discussion

Although it is currently accepted that the BM microenvironment contributes to AML development and chemoresistance, the underlying cellular and molecular mediators remain largely unknown. Notably, we and others have found that bulk BM stromal cells are reduced in AML, raising the question of which remaining niche cell populations promote leukemogenesis and chemoresistance (Baryawno et al., 2019; Méndez-Ferrer et al., 2020). Our current study has evaluated the role of nestin^+^ BMSCs in AML development and response to standard chemotherapy. The results suggest that nestin^+^ BMSCs provide LSCs with increased bioenergetics and GSH-related ROS detoxifying tools, contributing to AML development and chemoresistance.

Several *in vitro* studies have suggested functional alterations of BMSCs in AML, such as reduced proliferation (Corradi et al., 2018; Desbourdes et al., 2017; Yehudai-Resheff et al., 2019), increased apoptosis (Desbourdes et al., 2017), impaired differentiation and hematopoietic supporting activity in culture (Doron et al., 2019; Geyh et al., 2016), inflammatory prolife (Diaz de la Guardia et al., 2017; Forte et al., 2017; Jacamo et al., 2017; Kim et al., 2015; Kuett et al., 2015; Reikvam et al., 2015; Ruvolo et al., 2018; von der Heide et al., 2017), increased support of AML cells (Ben-Batalla et al., 2013; Brenner et al., 2017; Kornblau et al., 2018; Wu et al., 2018), and AML protection from chemotherapy through increased Notch (Takam Kamga et al., 2016) or Wnt (Lane et al., 2011) signaling and apoptosis inhibition (Carter et al., 2016, 2019). However, whether these or other BMSC alterations play a critical role in AML *in vivo* has remained unclear.

Unlike the bulk of BM stromal cells, nestin^+^ BMSCs were not diminished in AML patients or in iMLL-AF9 AML mouse model. These results are in agreement with findings in a different retrovirally expressed MLL-AF9 mouse model (Hanoun et al., 2014) and contrast with findings in myeloproliferative neoplasms (Arranz et al., 2014; Drexler et al., 2019), suggesting that the same niche cells exhibit different alterations and roles in various myeloid malignancies. Therefore, any strategy targeting the microenvironment should probably take into account the type and/or stage of malignancy.

Enforced ~50% reduction of nestin^+^ cells (*Nes-cre*^*ERT2*^*;iDTA*) caused a similar decrease of HSCs in non-leukemic mice. In contrast, *in vivo* nestin^+^ cell depletion in primary AML mice significantly decreased leukemia burden and extended mouse survival. Moreover, nestin^+^ cell depletion in chimeric mice carrying AML cells and WT cells did not affect normal hematopoietic progenitors but selectively decreased leukemia burden. These results suggest that the HSC niche function of nestin^+^ cells is altered in AML to support leukemic cells.

Different studies have shown that AML cells rely on OXPHOS for chemotherapy resistance (Baccelli et al., 2019; Farge et al., 2017; Jacque et al., 2015; Lagadinou et al., 2013; Molina et al., 2018; Pollyea et al., 2018). Among different substrates, AML cells can use fatty acids as biofuel (Farge et al., 2017). Reduced prolyl hydroxylase 3, which inhibits fatty acid oxidation (German et al., 2016), and increased fatty acid-binding protein 4 (Shafat et al., 2017) allow AML cells utilize fatty acids as substrates for OXPHOS. In fact, pharmacological inhibition of fatty acid oxidation can sensitize human AML cells to apoptosis induction (Samudio et al., 2010). The metabolic crosstalk between BMSCs and AML cells was investigated here using a novel coculture system, where human/murine BMSCs grown as non-adherent mesenspheres protected AML cells from chemotherapy-derived ROS-induced cell death, more efficiently than plastic-adherent BMSCs. In coculture with mesenspheres, AML cells exhibited increased OXPHOS, TCA cycle, and ATP production, confirming that BMSCs increase the bioenergetic capacity of AML cells.

Mitochondrial transfer from mesenspheres to leukemic blasts increased upon chemotherapy, in agreement with previous studies using plastic-adherent cells (Marlein et al., 2017; Moschoi et al., 2016). Despite the high ROS levels in AML cells (Li et al., 2011) and the uptake of ROS-generating mitochondria from BMSCs, ROS decreased (instead of increasing) in AraC-treated leukemic blasts cocultured with mesenspheres. The reason appears to be an augmented antioxidant capacity conferred by BMSCs.

A comprehensive comparison of transcriptomic changes in nestin^+^ BMSCs in AML with those genes differentially expressed in leukemic LK^lo^ cells of mice with/without nestin^+^ cell confirmed OXPHOS and mitochondrial-related pathways in this metabolic reprogramming and suggested candidate antioxidant mechanisms explaining BMSC protection from excessive ROS. This was particularly evident after AraC exposure, when proteomic analysis revealed a significant increase of GSH-related antioxidant proteins in coculture. In fact, GSH increased in AraC-treated AML cells cocultured with mesenspheres and was maximal in those leukemic blasts that had uptaken mitochondria from BMSCs. Interestingly, a TCA cycle metabolite that was increased in AML cells cocultured with BMSCs—fumarate (Figure 3K)—has been shown to bind to and activate Gpx1 to oxidize GSH and protect cells from ROS-induced cell death (Jin et al., 2015). Therefore, BMSC-dependent increase in TCA cycle intermediates (Figure 3K), *Gpx1* mRNA expression (Figure 5C) and GSH recycling (Figures 5 and 6) appear to converge upon increased Gpx-mediated AML protection from chemotherapy (Figure 6D). This possibility is supported by clinical data suggesting that mRNA expression of GSH-related antioxidant genes (including *GSS*, *GPX4*, or *GSTA1*) correlated with poor overall survival in human AML (Cancer Genome Atlas Research et al., 2013) (Figures S4B–S4E). Furthermore, Gpx1 inhibition with MSA abrogated the chemoprotective effect of BMSCs *in vitro*, whereas γ-GCS inhibition with BSO reduced GSH *in vivo* and synergized with standard chemotherapy. Combining BSO with standard chemotherapy allowed to eliminate chemoresistant leukemic cells, extend mouse disease-free survival, and significantly reduce LSCs measured after transplantations *in vivo*.

Consistent with our studies, depletion of cysteine has been recently shown to compromise LSC survival by inhibiting electron transport complex II (Jones et al., 2019). Along this line, targeting GSH metabolism has been suggested as a promising avenue to eradicate human AML (Gregory et al., 2019; Pei et al., 2013). As a novel aspect, our study suggests that BMSCs play a key role in GSH-dependent AML resistance. Adding up to our results, these studies suggest that cysteine-GSH might represent a key vulnerability axis in AML. Notably, the GSH system has been shown to critically regulate cancer cell survival in other malignancies. In chronic (Zhang et al., 2012) or acute (Boutter et al., 2014) lymphoblastic leukemia, BMSCs (but not cancer cells) effectively import cystine and convert it to cysteine, which is then secreted by BMSCs and is uptaken by cancer cells to generate GSH (Zhang et al., 2012). In summary, our results add further support to the relevance of GSH-mediated detoxification for AML chemoresistance and uncover a critical contribution from BMSCs to AML metabolic adaptation and survival *in vivo*.

### Limitations of Study

Although this study demonstrates a key role for NESTIN^+^ BMSC-dependent GSH and GPX in antioxidant protection of leukemic cells against chemotherapy, it does not exclude the possibility that other antioxidant pathways and cell types contribute to buffer excessive ROS in AML. Similarly, while the data clearly involve NESTIN^+^ cells in antioxidant protection, *in vivo* BSO treatment might deplete GSH in various cell types contributing to the final outcome—a synergistic effect to reduce LSCs when combined with standard chemotherapy mimicking clinical AML induction therapy.

## STAR★Methods

### Key Resources Table

**Table:** 

REAGENT or RESOURCE	SOURCE	IDENTIFIER
**Antibodies**		

CD3ε (145-2C11)	TONBO Biosciences	Cat#65-0031-U100; RRID: AB_2810847
Ly-6G (RB6-(C5)	BioLegend	Cat#127623; RRID: AB_10645331
sca-1 (E13-161.7)	BioLegend	Cat#108128; RRID: AB_2563064
Lineage Cocktail antibody	BD Biosciences	Cat#559971; RRID: AB_10053179
c-kit (2B8)	eBioscience	Cat#11-1171-81; RRID: AB_465185
CD150 (TC15-12F12.2)	BioLegend	Cat#115927; RRID: AB_11204248
Brilliant Violet 510™ Streptavidin	BioLegend	Cat#405233; RRID: AB_2810848
CD45.1 (A20)	BD Biosciences	Cat#560578; RRID: AB_1727488
CD45.2 (104)	TONBO Biosciences	Cat#20-0454; RRID: AB_2621576
Alexa Fluor 488 Sreptavidin-conjugated antibody	Invitrogen	Cat#S32354; RRID: AB_2315383
Mouse Anti-Human CD34 Clone 581 (RUO)	BD Biosciences	Cat# 555824, RRID: AB_398614
B220 (RA3-6B2)	BD Biosciences	Cat#553088; RRID: AB_394618
CD11b (M1/70)	BioLegend	Cat#101208; RRID: AB_312791
Biotin Rat Anti-Mouse CD31 Clone MEC 13.3	BD Biosciences	Cat#553371; RRID: AB_394817
Biotin Rat Anti-Mouse TER-119/Erythroid Cells Clone TER-119 (RUO)	BD Biosciences	Cat#553672; RRID: AB_394985

**Biological Samples**		

Human acute myeloid leukemia specimens	Cambridge University Hospitals, Cambridge, UK	REC 07-MRE05-44
Human acute myeloid leukemia specimens	University Hospital Sant’Orsola-Malpighi, Bologna, Italy	94/2016/O/Tess

**Chemicals, Peptides, and Recombinant Proteins**		

Fetal Bovine Serum, charcoal stripped	GIBCO	Cat#12676029
Cytarabine	Cayman Chemical	Cat#CAY16069
Collagenase Type I (0.25%)	Stem Cell Technologies	Cat#07902
Doxorubicin	Sigma	Cat#D1515
Lympholyte®-M Cell Separation Media	Cedarlane	Cat#CL5031
DMEM, high glucose, GlutaMAX™ Supplement, pyruvate	Thermo Fisher Scientific	Cat#31966021
RPMI Medium 1640 (1X) no phenol red	Thermo Fisher Scientific	Cat#11835-063
Murine IL-6	PeproTech	Cat#216-16-50
Murine SCF	PeproTech	Cat#216-16-50
Murine IL3	PeproTech	Cat#213-13-100
human PDGF-AA	PeproTech	Cat#100-13A-100
Human Oncostatin M (227 a.a.)	PeproTech	Cat#300-10T
Human IL-6	PeproTech	Cat#200-06-50
Human FGF-basic (154 a.a.)	PeproTech	Cat#100-18B
Poly(2-hydroxyethyl methacrylate)	Sigma-Aldrich	Cat#P3932
Human EGF	PeproTech	Cat#AF-100-15
Human IGF-I	PeproTech	Cat#100-11
B-27 Supplement (50X), serum free-10 mL	N/A	N/A
N-2 Supplement (100X)-5 mL	N/A	N/A
Trypsin (0.25%), phenol red	Gibco	Cat#15050065
DOXYCYCLINE HYCLATE, =98% (HPLC)	Sigma-Aldrich	Cat#D9891-25G
Penicillin-Streptomycin	Thermo Fisher Scientific	Cat#15140122
Corning™ Cell-Tak Cell and Tissue Adhesive	Fisher Scientific Ltd	Cat#CB40240
Dihydrorhodamine 123	Invitrogen	Cat#D23806
BODIPY™ 581/591 C11 (Lipid Peroxidation Sensor)	Invitrogen	Cat#D3861
Monochlorobimane (mBCI)	Invitrogen	Cat#M1381MP
MitoTracker™ Red CMXRos	Invitrogen	Cat#M7512
MitoTracker™ Green FM	Invitrogen	Cat#M7514
DAPI	Sigma-Aldrich	Cat#9542
Mercaptosuccinic acid	Sigma-Aldrich	Cat#M6182
DMSO	Sigma-Aldrich	Cat#D5879
buthionine sulfoximine (BSO)	N/A	Cat#NSC 326231
DTAB	Sigma-Aldrich	Cat# D8638
0.5M Trizma® base	Sigma-Aldrich	Cat#T1503

**Critical Commercial Assays**		

XF Cell Mito Stress Test	Agilent Technologies	Cat#103015-100
Seahorse XF Glycolytic Rate Assay	Agilent Technologies	Cat#103344-100
Pacific Blue™ Annexin V/SYTOX™ AADvanced™ Apoptosis Kit	Invitrogen	Cat#A35136
High-Capacity cDNA Reverse Transcription kit	Applied Biosystems	Cat#4368814
PowerUp SYBR Green Master Mix	Applied Biosystems	Cat#A25742
EasySep™ Human CD271 Positive Selection Kit	StemCell technologies	Cat#17849
ABC amplification kit	Vector Labs	Cat#AK-5000
Dynabeads mRNA DIRECT Kit	Thermo Fisher Scientific	Cat#61012
NADP/NADPH Glo Assay	Promega	Cat#G9081
Clit iT protein enrichment kit	Thermo Fisher Scientific (Life Technologies)	Cat#C10416
Arcturus Picopure RNA isolation kit	Applied Biosystems	Cat#KIT0204

**Deposited Data**		

TCGA dataset	cBioPortal (Cerami et al., 2012)	(Cancer Genome Atlas Research et al., 2013)
Proteomics data derived from this study	This study	Peptide Atlas (http://www.peptideatlas.org/repository/)Accession code: PASS01476
RNAseq data from nestin^+^ BMSCs obtained from leukemic *iMLL-AF9* and normal mice	This study	Gene Expression Omnibus (GEO), GSE140135
RNAseq data from leukemic BM lin^-^ckit^lo^ cells obtained from mice with/without nestin^+^ cell depletion	This study	Gene Expression Omnibus (GEO), GSE140207

**Experimental Models: Cell Lines**		

bEnd.3 [BEND3]	ATCC	Cat#CRL-2299

**Experimental Models: Organisms/Strains**		

congenic CD45.1 C57BL/6 mice	Charles River Laboratories	N/A
congenic CD45.2 C57BL/6 mice	Charles River Laboratories	N/A
*rtTA;MLL-AF9* mice		Stavropoulou et al., 2016
*Nes-cre*^*ERT2*^ mice	Gift from G. Fishell	Balordi and Fishell, 2007
*Nes-*GFP mice	Gift from G. Enikolopov	Mignone et al., 2004
*R26lacZbpA*^*flox*^*DTA* mice	Gift from D. Riethmacher	Brockschnieder et al., 2006

**Oligonucleotides**		

mouse *Gapdh* forward: GCATGGCCTTCCGTGTTC, reverse: CTGCTTCACCACCTTCTTGAT	Sigma-Aldrich	N/A
mouse *Gclc* forward: AGGCTCTCTGCACCATCACT, reverse: CTCTGGGTTGGGTCTGTGTT	Sigma-Aldrich	N/A
mouse *Gsr* forward: GCTATGCAACATTCGCAGATG, reverse: AGCGGTAAACTTTTTCCCATT	Sigma-Aldrich	N/A
mouse *Gpx1* forward: GGTTCGAGCCCAATTTTACA, reverse: CATTCCGCAGGAAGGTAAAG	Sigma-Aldrich	N/A
mouse *Gpx3* forward: ACAATTGTCCCAGTGTGTGCAT, reverse: TGGACCATCCCTGGGTTTC	Sigma-Aldrich	N/A
mouse *Actb* forward: TGGCGCTTTTGACTCAGGAT, reverse: GGGATGTTTGCTCCAACCAA	Sigma-Aldrich	N/A
mouse *Trx1* forward: ATGGTGAAGCTGATCGAGAGC, reverse: GGCATATTCAGTAATAGAGGC	Sigma-Aldrich	N/A
mouse *Trx2* forward: GCTAGAGAAGATGGTCGCCAAGCAGCA, reverse: TCCTCGTCCTTGATCCCCACAAACTTG	Sigma-Aldrich	N/A
mouse *Txnrd2* forward: GCTGGGCCTGCACTTCCT, reverse: ATGCACAGGTGATGCAGACAG	Sigma-Aldrich	N/A
mouse *Txnrd1* forward: CAAATTTGACAAGAAAGTGCTG, reverse: AGTCATGCTTCACTGTGTCTTC	Sigma-Aldrich	N/A
mouse *Sod1* forward: GTGATTGGGATTGCGCAGTA, reverse: TGGTTTGAGGGTAGCAGATGAGT	Sigma-Aldrich	N/A
mouse *Sod2* forward: TTAACGCGCAGATCATGCA, reverse: GGTGGCGTTGAGATTGTTCA	Sigma-Aldrich	N/A

**Software and Algorithms**		

Kaluza Analysis Software	Beckman Coulter	GalliosTM Kaluza, RRID: SCR_016700
GraphPad Prism v.8	GraphPad Software	GraphPad Prism, RRID: SCR_002798
Seahorse Wave v.2.2.0	Agilent	Seahorse Wave, RRID: SCR_014526
FlowJo	BD Biosciences	FlowJo, RRID: SCR_008520
Thermo Xcalibur	Thermo Fisher Scientific	Thermo Xcalibur, RRID: SCR_014593
TopHat v2.1.0	Johns Hopkins University, University of Washington and University of Maryland	TopHat, RRID: SCR_013035
R Project for Statistical Computing	R Foundation	R Project for Statistical Computing, RRID:SCR_001905
Proteome Discoverer v.2.1.0.81	Thermo Fisher Scientific	Proteome Discoverer, RRID: SCR_014477

### Resource Availability

#### Lead Contact

Further information and requests for resources and reagents should be addressed to the Lead Contact, Simon Mendez-Ferrer (sm2116@cam.ac.uk).

#### Materials Availability

New unique materials were not generated in the course of this study.

#### Data and Code Availability

All proteomics data derived from this study are deposited in Peptide Atlas (http://www.peptideatlas.org/repository/) and are accessible through the accession number PASS01476. The RNAseq data from nestin^+^ BMSCs obtained from leukemic *iMLL-AF9* and normal mice has been deposited in GEO under the accession number GSE140135. The RNAseq data from leukemic BM lin^-^ckit^lo^ cells obtained from mice with/without nestin^+^ cell depletion has been deposited in GEO under the accession number GSE140207. The human AML mRNA expression analysis and associated overall survival Kaplan-Meier estimate are accessible in the TCGA dataset (Cancer Genome Atlas Research et al., 2013) through the cBioPortal (Cerami et al., 2012). All the other data supporting the findings of this study are available within the article and its supplementary information files and from the lead contact author upon reasonable request.

### Experimental Model and Subject Details

#### Patient Samples

Human AML mononuclear cells were obtained from BM or peripheral blood of patients. Informed consent was obtained in accordance with the Declaration of Helsinki, and the study was conducted under local ethical approval (REC 07-MRE05-44 and 94/2016/O/Tess). Archival BM biopsies of patients, who consented to the use of their left-over tissue also for research purposes, from the Institute of Medical Genetics and Pathology at the University Hospital of Basel were utilized for purposes of the study according to the regulations of the safety laws of the canton Basel and the Swiss Federal Act on Research involving Human Beings; handling with probes was approved by the ethics committee of Northwestern Switzerland (EKNZ 2014-252). Samples were derived from 45 females and 49 males with a mean age of 60±19 years. Sample size was calculated using α = 0.05 and power 85%. No association was found between gender or age and NESTIN+ niches.

#### Mouse Models

8-12 week-old male or female (age and sex-matched) mice were used for experiments. Since no correlation was found between sex and NESTIN^+^ niches, both male and female mice were used in this study. Doxycycline-inducible *rtTA;MLL-AF9* mice, CD45.2 and CD45.1 C57BL/6J mice (Jackson Laboratories) were used in this study. Mice were housed in specific pathogen free facilities in individually ventilated cages under 12h light/darkness cycles and controlled temperature (19-23)°C and humidity (55±10%) with free access to standard rodent chow (SafeDiet R105-25). Mice were housed in IVC cages, all diet was irradiated and cages/bedding/environmental enrichment was autoclaved. Full cage changes were performed in changing stations and any procedures are carried out in a CLII cabinet. The Health Monitoring Surveillance Program consisted of the microbiology analysis of mouse sentinels and contact animals following the FELASA recommendations. Every quarterly period, sentinels and contact animals of the rack were bled for serology and tested for the agents recommended by FELASA (http://www.felasa.eu/recommendations/recommendation/recommendations-for-health-monitoring-of-rodent-and-rabbit-colonies/). Once a year, microbiology status of the contact animals is analyzed by PCR technique using feces and fur/oral swabs; and all sentinels (at the end of the 9-12 months) are euthanized for bacteriology, parasitology and macro-micro histopathological study (if required) by the CNIC’s microbiology and histology laboratories. At Cambridge University, FELASA PLUS screening was performed annually and Klebsiella spp was analyzed as an additional agent. All screenings revealed no significant findings at either Establishment. All animal procedures conformed to the United Kingdom Home Office regulations (PPL 70/8406 and P0242B783) and EU Directive 2010/63EU and Recommendation 2007/526/EC regarding the protection of animals used for experimental and other scientific purposes, enforced in Spanish law under Real Decreto 1201/2005, and were approved by the local ethics committees and the Animal Protection Area of Cambridge (UK) and the Comunidad Autónoma de Madrid (PROEX 154/14).

Generation and full characterization of *rtTA;MLL-AF9* mice have been described elsewhere (Stavropoulou et al., 2016). Briefly, this transgenic doxycycline-inducible strain expresses rtTA in the ROSA26 locus and the MLL-AF9 fusion of human origin under the control of the tetracycline-response element. Upon induction, both the primary *rtTA;MLL-AF9* (*iMLL-AF9*) mice and WT recipients of *iMLL-AF9* BM cells develop a similar type of AML (M4/5) which recapitulates the human disease. In order to follow the development of normal and leukemic hematopoiesis simultaneously in the same animals, we competitively transplanted lethally irradiated recipients (12 Gy whole body irradiation, split dose 6.0 + 6.0 Gy, 3 h apart) with non-induced, *iMLL-AF9* BM (10^6^ cells) and WT BM (10^6^ cells). The CD45.1/2 epitope combination was commonly as CD45.2 for *iMLL-AF9* and CD45.1 for WT but it was reversed in some experiments, with no appreciable differences. Then after 2 weeks under antibiotics, mice were kept with doxycycline (1% in 5% sucrose water or pellet food 400 mg/kg) to induce transgene expression. Disease progression was monitored every week in tail peripheral blood samples using an automated blood counter. Mice were considered as leukemic once peripheric white blood counts were over 15 x 10^3^/ul associated with increased percentages in the monocyte and granulocyte cell populations. Mice were sacrificed at the appropriate endpoints. BM, spleen and blood were analyzed by cell counts, histology and flow cytometry, and cells were used for functional studies as required.

Different manipulations of the BM microenvironment were achieved through the use of diverse recipient strains. In order to selectively deplete Nes+ cells, Nes-cre^ERT2^ mice (Balordi and Fishell, 2007) were crossed with a mouse line harboring a Cre-inducible diphtheria toxin gene (iDTA) (Brockschnieder et al., 2006), yielding *Nes-cre*^*ERT2*^;R26lacZbpA^flox^DTA mice (abbreviated as *Nes-cre*^*ERT2*^;*iDTA*). Cre^ERT2^ recombinase was activated by tamoxifen treatment (Sigma) (140mg/kg of tamoxifen, 14mg/ml solution in corn oil, 3 times/week i.p. on alternate days, typically starting one week after the initiation of doxycycline induction). For combined Nes+ cell depletion and chemotherapy administration, *Nes-cre*^*ERT2*^;*iDTA* and control mice were simultaneously treated with tamoxifen (140 mg/kg, i.p., 3 doses on alternate days) and AraC (5 daily doses of 100 mg/kg, i.p.), starting 2 weeks after MLL-AF9 induction. For acute response to AraC, AraC was injected 24 hours after a single tamoxifen injection and mice sacrificed for analysis 24 hours later. For analysis of targeting the GSH antioxidant pathway in vivo, at the disease onset, mice were randomised into two treatment groups, one given chemotherapy (5+3 regimen with 100mg/kg/d AraC and 3mg/kg/d doxorubicin for 3 days followed by 100mg/kg/d AraC for 2 days, i.p.) once daily (group Chemo only) and the other chemotherapy in combination with 400mg/kg BSO (i.p., group Chemo+BSO). After 5 days, daily BSO injections continued for 5 more days, whereas the control group received vehicle injections. Mice were sacrificed after 11 days for immunophenotypic analysis or at the appropriate endpoints. BM, spleen and blood were analyzed by cell counts and flow cytometry.

### Method Details

#### Immunohistochemistry of Human Samples

For NESTIN/CD34 immunohistochemistry of human BM samples, 12 control BM biopsies (2 from healthy donors, 2 from patients with reactive peripheral leukocytosis, and 8 performed for lymphoma staging, but unaffected by lymphoma) and 37 AML (5 MLL-AF9^+^ and 56 with different cytogenetics) were stained for NESTIN applying the monoclonal antibody 10C2 from AbD Serotec (OBT1610) at a dilution of 1:50 using an automated immunostainer (Benchmark, Ventana/Roche). Antigen retrieval was achieved by cell conditioning (CC1 from Ventana/Roche) treatment for 60min. Incubation for 60min, signal amplification and visualization (amplifier and chromogen ultraview universal diaminobenzidine from Ventana/Roche) followed. For NESTIN and CD34 double immunostainings, the anti-CD34 monoclonal antibody QBEnd/10 (Ventana/Roche 790-2927) was used after NESTIN detection and visualized using an alternative chromogenic detection kit (basic aminoethylcarbazole from Ventana/Roche).

#### Cell Isolation and Culture

Mesenspheres were cultured from mouse primary BM cells in the following way. Clean mouse bones were crushed in a mortar with 2 ml of a solution containing Collagenase Type I (0.25%) (Stem Cell Technologies). The suspension was incubated for 45 minutes at 37 °C in agitation. After addition of PBS+ 2% FBS and passage through a 40 μm cell strainer, erythrocytes were lysed by incubation on ice with RBC Lysis Buffer. After this, erythroid, endothelial and hematopoietic cells were removed by magnetic depletion after incubation with biotin-conjugated primary antibodies against CD45, Ter119 and CD31 (BD Biosciences, 1:100) and subsequent incubation with streptavidin-conjugated magnetic beads (BD Biosciences). For sphere formation, the cells immunomagnetically depleted of hematopoietic (CD45+), erythroid (Ter119+) and endothelial (CD31+) cells were plated at low density (<500,000 cells/cm2) in ultralow-adherence 35 mm dishes (StemCell Technologies) after treatment with Poly-Hema (Sigma). The growth medium for spheres contained 0.1 mM β-mercaptoethanol; 1% nonessential amino acids (Sigma); 1% N2 and 2% B27 supplements (Invitrogen); recombinant human fibroblast growth factor (FGF)-basic, recombinant human epidermal growth factor (EGF), recombinant human platelet-derived growth factor (PDGF-AA), recombinant human oncostatin M (227 aa OSM, 20 ng/ml) and recombinant human IGF-1 (40 ng/ml; Peprotech) in Dulbecco’s modified Eagle’s medium (DMEM)/F12 (1:1) / human endothelial (1:2) serum-free medium (Invitrogen). Mesensphere medium was supplemented with 15% CEE prepared as described previously (Pajtler et al., 2010) with minor modifications. Briefly, fertilized chicken eggs were incubated for 11 days at 38°C in a humidified incubator. Embryos were washed with DMEM (Invitrogen), macerated by passage through a 50 ml syringe and diluted 1:1 in the same medium. Hyaluronidase (2 mg, Sigma) was added to each 50 ml tube and incubated 1h at 4°C with agitation. Following 6 h ultracentrifugation (3x10^4^ *g*) at 4°C was decanted and filtered with 0.45 μm and 0.22 μm sterile filters (Millipore). Aliquots were stored at -80°C until use. The cultures were incubated at 37°C with 5% CO_2_, 20% O_2_ in a water-jacketed incubator and left untouched for 1 week. Afterwards, half-medium changes were performed twice a week. For passage, spheres were enzymatically dissociated with 100μl Trypsin (EDTA-free) for 10 min at 37°C, applying mechanical dispersion every 10 min. The cells were washed with PBS once and replated with mesensphere medium in ultralow-adherence 35mm dishes (StemCell Technologies).

Human mesenspheres were isolated after magnetic CD271 cell selection using EasySep™ Human CD271 Positive Selection Kit (StemCell Technologies) from patients undergoing diagnostic BM aspiration and were grown as previously described (Isern et al., 2013) and similarly to mouse mesensphere culture described above.

Human AML CD34+ cells were isolated by magnetic cell selection using CD34 MicroBead Kit UltraPure (Miltenyi) after Bone Marrow (BM) or Peripheral Blood (PB) Mononuclear Cells (MNCs) density gradient centrifugation by Ficoll-Paque.

MLL-AF9 mouse leukemic blasts were isolated from bones of iMLL-AF9 mice and maintained in 6 well plates in RPMI 1640 (Invitrogen) without phenol red and supplemented with 10% charcoal-stripped FBS (Gibco), recombinant murine IL3, recombinant murine SCF and recombinant human IL-6 (10 ng/ml) (Peprotech), 1% Penicillin-Streptomycin, 1 ug/ml doxycycline at 37°C in a water-jacketed incubator with 5% CO_2_ and 20% O_2_. Cells were split every other day and seeded at 500,000 cells/ml.

We set up co-cultures systems with mesenspheres (~200 per ml) and leukemic blasts (250,000 cells/ml) for 24h in RPMI without phenol red and with charcoal-stripped FBS (Gibco) ± cytarabine (AraC, Cayman Chemical, 1μM) in flat-bottom 96-well low adherence tissue cultures plates (Costar) at 37°C in a water-jacketed incubator with 5% CO_2_ and 20% O_2_. Cultures were grown for 24h before flow-cytometry staining (apoptosis, ROS levels, lipid peroxidation).

#### NADP/NADPH Measurement

NADP/NADPH levels in cell were measured using the NADP/NADPH-Glo Bioluminescent Assay kit (Promega G9082) after sorting leukemic blasts from the cocultures as CD45^+^ cells. Briefly, after lysing cells in the recommended base solution with dodecyltrimethyl ammonium bromide (DTAB), the samples were split into separate wells for acid and base treatments (Trizma®±HCl). The treated samples were recorded for luminescence after incubation with NADP/NADPH-Glo™ Detection Reagent at room temperature for 40 min.

#### Seahorse Metabolic Extracellular Flux Profiling

Leukemic blasts (mono-cultured or co-cultured with mesenspheres) were seeded in 24-well plate as above described. After 24h incubation, cells were seeded (500,000 cells/well) in Seahorse 96-well plates coated with CellTak (BD Biosciences, San Jose, CA, USA) to facilitate attachment.

Cell Mito Stress Test (XF Cell Mito Stress Test Kit, Agilent Technologies) was performed following the standard protocol (Nicholls et al., 2010). Oxygen consumption rate (OCR) and Extracellular Acidification Rate (ECAR) were detected after injection of oligomycin (1 μM), Carbonyl cyanide-p-trifluoromethoxyphenylhydrazone (FCCP, 0.5 μM), and the combination of rotenone & antimycin (Rot/AA, 0.5 μM). OCR was measured with a XF96 analyzer and the Wave software (version 2.2.0) (Seahorse Bioscience).

#### Flow Cytometry and Fluorescent-Activated Cell Sorting (FACS)

Briefly, after co-culture experiments, cell suspension with both types of cells (leukemic blasts and mesenspheres) was centrifuged for 5 min at 300 x g and resuspended in appropriate volume (~1ml). Then, non-aggregated single cells (most of leukemic blasts) were filtered out by passing through Test Tube with Cell Strainer Snap Cap (35μm nylon mesh) (Corning™ Falcon™) in order to separate aggregated mesenspheres from leukemic blasts. After flipping the strainer onto a new tube to release all aggregate and after visually confirming that the aggregates have been rinsed off the filter, leukemic blasts (passed through the filter cap) were collected and washed. Finally, mesenspheres were treated with trypsin no-EDTA for 10 minutes at 37 °C and then were stained for stromal cell markers in selected experiments (e.g. CD105), whereas leukemic blasts were stained with specific markers (namely CD45, CD34) in order to gate/sort positive leukemic blasts (mainly CD45+) from negative stromal fraction for FACS experiments.

Leukemic blasts (monocultured or cocultured with BMSCs) were incubated with the appropriate dilution (2-5 μg/ml) of fluorescent antibody conjugates and were stained in PBS containing 2% FBS at 4°C, washed, and analyzed using a Gallios flow cytometer (Beckman Coulter, Miami Lakes, FL) and Kaluza software (Beckman Coulter).

For immunophenotyping of hematopoietic cell populations, bones (limbs, sternum and spine) were crushed in a mortar, filtered through a 40-μm mesh to obtain single cell suspensions, and depleted of red blood cells by lysis in 0.15 M NH_4_Cl for 10 min at 4°C. Blood samples were directly lysed in the same buffer for 10 min at room temperature. Cells (1-2 x 10^6^ cells/sample) were incubated with the appropriate dilution (2-5 μg/ml) of fluorescent antibody conjugates and 4',6-diamidino-2-phenylindole (DAPI) for dead cell exclusion, and analyzed on FACS Canto II, LSR Fortessa (BD Biosciences, Franklin Lakes, NJ) or Gallios flow cytometer (Beckman Coulter, Miami Lakes, FL). FlowJo and Kaluza softwares were used for data analysis. The following antibody conjugates were used: CD45.1-PE-Cy7 (A20), CD45.2-APC-Cy7 (104), B220-FITC (RA3-6B2), CD11b-PE (M1/70), CD3ε-PerCP-Cy5.5 (145-2C11), Sca1-PE (E13-161.7), CD34-FITC (RAM34), CD135/Flt3-APC (A2F10.1), and biotinylated lineage antibodies (CD11b, Gr-1, Ter119, B220, CD3ε), all from BD Biosciences; c-kit-PE-Cy7 or -APC (2B8), from eBioscience (San Diego, CA). Biotinylated antibodies were detected with APC-Cy7-conjugated streptavidin (BD Biosciences).

Intracellular reactive oxygen species (ROS) were detected by staining cells with dihydrorhodamine 123 (DHR123, Thermo Fisher) following the manufacturer’s recommendations. To assess lipid peroxidation, cells were incubated at a final concentration of 5 μM C11-BODIPY^581/591^ (Life Technologies-Invitrogen, Carlsbad, USA). In addition, the cell-permeant probe monochloromobimane (mBCI, Molecular Probes) was used for quantifying glutathione levels inside cells.

For the analysis of stromal cell populations, bones were directly crushed in 0.25% Collagenase Type I (Stem Cell Technologies) and incubated at 37°C for 45 min in a water bath. The resulting cell suspensions were filtered and lysed as above and incubated with CD45-PE, CD31-APC and Ter119-PE-Cy7 antibodies for 15-20 min at 4°C.

For cell sorting, BM samples were processed similarly, except that a lineage depletion step preceded the final staining. For lineage depletion, cells were first incubated with biotinylated lineage antibodies as above, followed by addition of streptavidin beads (BD Bioscience) and magnetic depletion. To discriminate between the leukemic blasts and mesenspheres in coculture, CD45 staining was used after filtration using cell strainer snap cap (35 μM). Populations of interest were separated in a FACS Aria cell sorter (BD Bioscience).

For the determination of apoptotic cells, samples were washed with PBS after surface antibody staining (if required) and subsequently stained with Annexin V-Pacific Blue and SYTOX AADvanced (Invitrogen, Life Technologies, Paisley, UK), following the manufacturer's instructions.

The transfer of mitochondria was evaluated using a green-fluorescent mitochondrial dye (Mitotracker Green FM) and a red-fluorescent dye (MitoTracker Red CMXRos) (Thermo FisherWaltham, MA USA). Briefly, leukemic blasts were stained with Mitotracker Green FM (100 ng) and the mesenspheres were separately stained with Mitotracker Red CMXRos (50 ng) for 20min at 37°C and the unbound dyes were removed by extensively washing. The leukemic blasts and the mesenspheres were seeded separately in medium for 24h at 37°C. Before coculture, both types of cells were washed twice, collected and counted. The transfer of mitochondria was evaluated by flow cytometry 24h after coculture.

#### Liquid Chromatography Coupled to Mass Spectrometry (LC-MS) for Metabolomics Analysis

Leukemic blasts (800,000 cells/ml) were cultured alone or in coculture with mesenspheres (250/ml) in normal media for 24h in the presence of AraC. All the cells were filtered using Falcon tube with cell strainer snap cap (Corning) to mechanically separate leukemic blasts from mesenspheres. Cells were washed three times with PBS and the extraction buffer (50% methanol, 30% acetonitrile, 20% water, all LC-MS grade) was added (1ml/4x10^6^ cells). Cell were incubated in dry ice for 15 min, collected, vigorously shaked for 15 min at 4°C and left 1h at -20°C. Samples were centrifuged at 13,000 rpm and supernatants were transferred to autosampler vials and stored at -80 °C until further analysis. To avoid bias due to machine drift, samples were randomized and processed blindly.

A Q Exactive mass spectrometer (Thermo Fisher Scientific) coupled to a Dionex U3000 UHPLC (Thermo Fisher Scientific) system was used to perform the LC-MS analysis. A Sequant ZIC-pHILIC column (150 x 2.1 mm, 5 μm) and guard column (20 x 2.1 mm, 5 μm) (Merck Millipore) were utilized for the chromatographic separation. The column oven temperature was maintained at 40°C. The mobile phase was composed of 20 mM ammonium carbonate and 0.1% ammonium hydroxide in water (solvent A), and acetonitrile (solvent B). The flow rate was set at 0.2 ml/min with the following gradient: 80% B for 2 min, linear decrease to 20% of B 15 min. Both solvents were then brought back to initial conditions and maintained for 8 min. The mass spectrometer was operated in full MS and polarity switching mode. XCalibur Qual Browser and XCalibur Quan Browser software (Thermo Fisher Scientific) were used to process and analyze the spectra. Ion intensities indicate raw (not normalized) values.

#### RNA Isolation and qPCR

RNA isolation was performed using Dynabeads® mRNA Purification Kit (Thermo Fisher Scientific 61012). Reverse transcription was performed using the High-Capacity cDNA Reverse Transcription kit (Applied Biosystems 4368814), following the manufacturer’s recommendations. qPCR was performed using the PowerUp SYBR Green Master Mix (Applied Biosystems A25742) and ABI PRISM® 7900HT Sequence Detection System.

The sequences of the primers used are detailed in the Key Resource Table.

#### RNAseq

For Nestin+ BMSC RNAseq, *Nes*-GFP^+^ cells were sorted from the BM of normal or iMLL-AF9^+^ mice as follows. Leukemia was as described above by providing doxycycline. Control mice were provided 5% sucrose in water as vehicle. A non-GFP mouse was used as a control to set up the gating strategy during sorting.

Bones from leukemic or non-leukemic mice were processed as described above and CD45^-^CD31^-^Ter119^-^DAPI^-^*Nes*-GFP^+^ cells were sorted and immediately frozen at -80°C. mRNA was obtained using the RNeasy Kit (Qiagen). Due to low cell number available, an amplification step was performed as previously described (Picelli et al., 2014). Briefly, the Smart-seq2 protocol was implemented with improved reverse transcription, template switching and preamplification to increase both yield and length of cDNA libraries generated from individual cells. Reads were aligned using TopHat version v2.1.0 (Kim et al., 2013) to genome build GRCm38/mm10.

Table S1 shows the analysis of the RNAseq data from Nestin^+^ BMSCs obtained from leukemic iMLL-AF9 and normal mice.

RNAseq of MLL-AF9^+^ lin^-^ ckit^lo^ sca1^-^ (LK^lo^) cells was performed after sorting these cells from the BM of *Nes-cre*^*ERT2*^*;iDTA* (N=8) or control (N=6) mice. Sorted cells were combined for a total of 3 biological replicates (each consisting of pooled LK^lo^ cells from from 2-3 mice). Total RNA was isolated using the Arcturus Picopure RNA Isolation Kit (Thermo Fisher Scientific). RNA was amplified and prepared for RNA-Seq using the Ovation RNA-Seq System v2 (NuGEN) following the manufacturer’s recommendations.

Table S2 shows the analysis of the RNAseq data from leukemic BM lin^-^ ckit^lo^ cells obtained from mice with/without nestin^+^ cell depletion.

The RNA sequencing library was prepared with the TruSeq RNA Sample Preparation v2 Kit (Illumina, San Diego, CA) to construct index-tagged cDNA. The quality, quantity and the size distribution of the Illumina libraries were determined using the DNA-1000 Kit (Agilent Bioanalyzer). Libraries were sequenced on the Genome Analyzer IIx (Illumina) following the standard RNA sequencing protocol with the TruSeq SBS Kit v5. Fastq files containing reads for each library were extracted and demultiplexed using Casava v1.8.2 pipeline.

Sequencing adaptor contaminations were removed from reads using cutadapt software tool (MIT) and the resulting reads were mapped and quantified on the transcriptome (NCBIM37 Ensembl gene-build 65) using RSEM v1.17 (Li and Dewey, 2011). Only genes with more than 2 counts per million in at least 2 samples were considered for statistical analysis. Data were then normalized and differential expression was assessed using the bioconductor package EdgeR (Robinson et al., 2010).

Expression data from 12337 genes was obtained and compared between BM LK^lo^ cells from mice with/without nestin^+^ cell depletion, using an empirical Bayes statistic for differential expression (moderated t-test), as implemented in limma package (R/Bioconductor). Genes with adjusted p-value ≤ 0.05 were considered to be differentially expressed between the two conditions. Additionally, in order to detect coordinated changes in sets of genes representing pathways or functional signatures, we performed gene-set enrichment analyses (GSEA) against the collection of 3144 curated gene sets available in the Molecular Signatures Database (http://www.broadinstitute.org/gsea/msigdb/index.jsp), including KEGG, Biocarta and Reactome pathways as well as a collection of gene expression signatures associated with chemical or genetic perturbations. Significance of gene set enrichment between the two conditions was assessed with GSEA software as previously described (Subramanian et al., 2005) (http://www.broadinstitute.org/gsea/index.jsp), using a weighted statistic, ranking by signal to noise ratio and 1000 gene-set permutations. Gene sets with FDR<0.05 were considered to exhibit the most significantly enrichment.

#### Quantitative Proteomic Analysis

##### Sample Preparation

Blasts and BMSCs (spheres) pellets were obtained by filtering the culture using 35μm cell strainer snap cap tubes to isolate the spheres (which did not pass the filter), following isolation of leukemic blasts as CD45^+^ DAPI^-^ cells. Different conditions included blasts cultured alone (n=2), blast cultured alone treated with AraC (n=3), blast co-cultured with spheres (n=3), blast co-cultured with spheres treated with AraC (n=3), spheres cultured alone (n=2), spheres cultured alone treated with AraC (n=2), spheres co-cultured with blasts (n=3) and spheres co-cultured with blasts treated with AraC (n=4). Each sample was generated by pooling cells from different cultures due to the scarcity of material for each of them. Pellets were extracted in Lysis buffer (50 mM Tris-HCl pH 7.5; 2% SDS, 10 mM TCEP (Tris(2-carboxyethyl) phosphine hydrochloride (TCEP)) by homogenizing the cells with vortex, boiling for 5 min, and incubating for 30 min at RT with agitation. Samples were centrifuged at 15,000 rpm for 15 min and protein concentration was determined using a Direct Detect IR spectrometer (Millipore).

##### Protein Digestion and Isobaric Labelling

For the quantitative differential analysis by LC-MS/MS using isobaric tags (TMT 10-plex), about 100 μg of total proteins were digested using the FASP protocol as previously described with minor modifications (Cardona et al., 2015). Briefly, proteins were diluted in 7 M urea in 0.1 M Tris-HCl (pH 8.5) (UA), and loaded onto 10 kDa centrifugal filter devices (NanoSep 10k Omega, Pall Life Sciences). Samples were washed into filters with UA, and proteins were then alkylated with 50 mM iodoacetamide (IAA) in UA for 30 min in the dark. Samples were washed three times with UA and three additional times with 50 mM ammonium bicarbonate. Proteins were digested overnight at 37°C with modified trypsin (Promega) in 50 mM ammonium bicarbonate at 30:1 protein:trypsin (w/w) ratio. The resulting peptides were eluted by centrifugation with 50 mM ammonium bicarbonate, and 0.5M sodium chloride. Trifluoroacetic acid (TFA) was added to a final concentration of 1% and the peptides were desalted onto C18 Oasis-HLB cartridges and dried-down for further analysis.

For stable isobaric labelling, the resulting tryptic peptides were dissolved in 30 μl of 100 mM Triethylammonium bicarbonate (TEAB) buffer, and the peptide concentration was determined by measuring amide bonds with the Direct Detect system (Millipore). Equal amounts of each peptide sample were labelled using the 10-plex TMT Reagents (Thermo Fisher) according to manufacturer's protocol. Peptides were labelled with TMT reagents previously reconstituted with 70 μl of acetonitrile, and after incubation at room temperature for 1h, reaction was stopped with 0.5% TFA, incubated for 15 min, and peptides were combined. Samples were concentrated in a Speed Vac, desalted onto C18 Oasis-HLB cartridges and dried-down for further analysis. For increasing proteome coverage, TMT-labelled samples were fractionated by high-pH reverse phase chromatography (High pH Reversed-Phase Peptide Fractionation Kit, Pierce) and concentrated as before.

##### Protein Identification

Labelled peptides were analyzed by LC-MS/MS using a C-18 reversed phase nano-column (75 μm I.D. x 50 cm, 2 μm particle size, Acclaim PepMap RSLC, 100 C18; Thermo Fisher Scientific) in a continuous acetonitrile gradient consisting of 0-30% B in 360 min, 50-90% B in 3 min (A= 0.1% formic acid; B=90% acetonitrile, 0.1% formic acid). A flow rate of 200 nL/min was used to elute peptides from the nano-column to an emitter nanospray needle for real time ionization and peptide fragmentation on an Orbitrap Fusion mass spectrometer (Thermo Fisher). An enhanced FT-resolution spectrum (resolution=70,000) followed by the MS/MS spectra from the Nth most intense parent ions were analyzed along the chromatographic run. Dynamic exclusion was set at 40s.

For peptide identification, all spectra were analyzed with Proteome Discoverer (version 2.1.0.81, Thermo Fisher Scientific) using SEQUEST-HT (Thermo Fisher Scientific). For database searching at the Uniprot database containing all sequences from mouse and contaminants (April 27, 2016; 48,644 entries), the parameters were selected as follows: trypsin digestion with 2 maximum missed cleavage sites, precursor and fragment mass tolerances of 2 Da and 0.02 Da, respectively. Carbamidomethyl cysteine (+57.021 Da) and TMT modifications (+229.162932 Da) at N-terminal and Lys residues were selected as fixed modifications, and methionine oxidation (+15.994915 Da) as dynamic modification.

#### Functional Protein Analysis

Functional protein analysis of the whole set of quantified proteins was performed using our algorithm, system biology triangle (SBT), developed specifically for the analysis of coordinated protein responses in high-throughput quantitative proteomics experiments (García-Marqués et al., 2016). This algorithm correlates the performance of a group of proteins inside of a category (biological process) in terms of their quantitative behavior (relative abundance); thus, changes can be detected in functional biological processes far beyond individual protein responses. Because of this coordinated behavior, a Z value is assigned to each category. Variations in the abundance of annotated functional categories were visualized by comparing the cumulative frequency (sigmoid) plots of the standardized variable with that of the normal distribution, as previously described (Isern et al., 2013). Briefly, differentially-enriched pathways were compared with the predicted cumulative normality plot of the standardized variable at the protein level for all proteins. Individual protein changes were also considered for further analysis.

Table S3 shows the analysis of the proteomic data from leukemic blasts and spheres in monoculture or coculture upon AraC treatment.

#### CBioPortal and TCGA

The human AML mRNA expression analysis and associated overall survival Kaplan-Meier estimate were obtained from the TCGA dataset (Cancer Genome Atlas Research et al., 2013) in cBioPortal (Cerami et al., 2012).

### Quantification and Statistical Analysis

The number of NESTIN^+^ perivascular niches (either single cells or clusters of up to 3 cells) was blindly scored in human BM samples. An average area of 7.2mm^2^ was evaluated for each case.

For qPCR, the expression level of each gene was determined by using the absolute quantification standard curve method. All values were normalized with *Actin* as endogenous housekeeping gene.

For RNAseq, gene expression was quantified using featureCounts version 1.5.0 (Liao et al., 2014) with annotation from Ensembl Release 86 (Yates et al., 2016). Normalization and differential expression were performed using Deseq2 version 1.14.1 (Love et al., 2014), using R version 3.3.3. Gene ontology enrichment was performed using the R package goseq (Young et al., 2010) for significantly differentially expressed genes (padj > 0.05). KEGG pathway enrichment was performed using the R package SPIA (Tarca et al., 2009) or DAVID (Huang et al., 2007).

For LC-MS/MS peptide identification was performed using the probability ratio method (Martínez-Bartolomé et al., 2008) and false discovery rate (FDR) was calculated using inverted databases, and the refined method (Navarro and Vazquez, 2009) with an additional filtering for precursor mass tolerance of 10 ppm (Bonzon-Kulichenko et al., 2015). Identified peptides with an FDR equal or lower than 1% FDR were used to quantify the relative abundance of each protein from reporter ion intensities, and statistical analysis of quantitative data were performed using the WSPP statistical model previously described (Navarro et al., 2014). Briefly, in this model protein log_2_-ratios are expressed as standardized variables, i.e., in units of standard deviation according to their estimated variances (Zq values).

Statistical parametric analyses were used after confirming that values followed a normal distribution. Student’s t test was used for 2 group comparison. One-Way ANOVA and Bonferroni comparisons were used for multigroup comparison. Logrank test was used for survival analysis. p values less than 0.05 were considered statistically significant. Statistical analyses and graphics were carried out with GraphPad Prism 8 software and Microsoft Excel.

## Supplemental Information

Document S1. Figures S1–S4

Table S1. Analysis of the RNA-Seq Data from Nestin+ BMSCs Obtained from Leukemic iMLL-AF9 and Normal Mice, Related to Figure 4

Table S2. Analysis of the RNA-Seq Data from Leukemic BM lin− ckitlo Cells Obtained from Mice with/without Nestin+ Cell Depletion, Related to Figure 4

Table S3. Analysis of the Proteomic Data from Leukemic Blasts and Spheres in Monoculture or Coculture upon AraC Treatment, Related to Figure 4

Document S2. Article plus Supplemental Information
