# Supplementary material for: Bone Marrow Mesenchymal Stem Cells Support Acute Myeloid Leukemia Bioenergetics and Enhance Antioxidant Defense and Escape from Chemotherapy
Source: Cell Metab. 2020 Nov 3;32(5):829–843.e9. doi: 10.1016/j.cmet.2020.09.001 (PMC7658808; doi:10.1016/j.cmet.2020.09.001)
Supplement: Document S1. Figures S1–S4 [file mmc1.pdf]

Cell Metabolism, Volume *32*

Supplemental Information

Bone Marrow Mesenchymal Stem Cells Support Acute Myeloid Leukemia Bioenergetics and Enhance Antioxidant Defense and Escape from Chemotherapy

Dorian Forte, María García-Fernández, Abel Sánchez-Aguilera, Vaia Stavropoulou, Claire Fielding, Daniel Martín-Pérez, Juan Antonio López, Ana S.H. Costa, Laura Tronci, Efterpi Nikitopoulou, Michael Barber, Paolo Gallipoli, Ludovica Marando, Carlos López Fernández de Castillejo, Alexandar Tzankov, Sabine Dietmann, Michele Cavo, Lucia Catani, Antonio Curti, Jesús Vázquez, Christian Frezza, Brian J. Huntly, Juerg Schwaller, and Simón Méndez-Ferrer

Cell Metabolism, Volume *32*

Supplemental Information

Bone Marrow Mesenchymal Stem Cells Support Acute Myeloid Leukemia Bioenergetics and Enhance Antioxidant Defense and Escape from Chemotherapy

Dorian Forte, María García-Fernández, Abel Sánchez-Aguilera, Vaia Stavropoulou, Claire Fielding, Daniel Martín-Pérez, Juan Antonio López, Ana S.H. Costa, Laura Tronci, Efterpi Nikitopoulou, Michael Barber, Paolo Gallipoli, Ludovica Marando, Carlos López Fernández de Castillejo, Alexandar Tzankov, Sabine Dietmann, Michele Cavo, Lucia Catani, Antonio Curti, Jesús Vázquez, Christian Frezza, Brian J. Huntly, Juerg Schwaller, and Simón Méndez-Ferrer

SUPPLEMENTAL INFORMATION

Bone Marrow Mesenchymal Stem Cells Support Acute Myeloid Leukemia Bioenergetics and Enhance Antioxidant Defense and Escape from Chemotherapy

Dorian Forte[1-3,*], María García-Fernández[1-3,*], Abel Sánchez-Aguilera[4,*], Vaia Stavropoulou[5], Claire Fielding[1-3], Daniel Martín-Pérez[4], Juan Antonio López[4,6], Ana S.H. Costa[7], Laura Tronci[7], Efterpi Nikitopoulou[7], Michael Barber[1], Paolo Gallipoli[1-2], Ludovica Marando[1-2], Carlos López Fernández de Castillejo[4], Alexandar Tzankov[8], Sabine Dietmann[1], Michele Cavo[9], Lucia Catani[9], Antonio Curti[10], Jesús Vázquez[4,6], Christian Frezza[7], Brian J. Huntly[1-2], Juerg Schwaller[5,#] and Simón Méndez-Ferrer[1-4,#]

[1]Wellcome-MRC Cambridge Stem Cell Institute, CB2 0AW Cambridge, United Kingdom

[2]Department of Haematology, University of Cambridge, CB2 0AW Cambridge, United Kingdom

[3]National Health Service Blood and Transplant, CB2 0PT Cambridge, United Kingdom

[4]Centro Nacional de Investigaciones Cardiovasculares (CNIC), 28029 Madrid, Spain

[5]University Children's Hospital and Department of Biomedicine (DBM), University of Basel, CH-4031 Basel, Switzerland

[6]CIBER de Enfermedades Cardiovasculares (CIBERCV), Spain

[7]MRC Cancer Unit, University of Cambridge, CB2 0XZ Cambridge, United Kingdom

[8]Institute of Pathology, University Hospital Basel, CH-4031 Basel, Switzerland

[9]Department of Experimental, Diagnostic and Specialty Medicine (DIMES), Institute of Hematology "L. and A. Seràgnoli", University of Bologna, 40138 Bologna, Italy

[10]Institute of Hematology "L. and A. Seràgnoli", Department of Hematology and Oncology, University Hospital Sant'Orsola-Malpighi, 40138 Bologna, Italy

*These authors contributed equally

[#]Correspondence: S.M.-F. (sm2116@cam.ac.uk) or J.S. (j.schwaller@unibas.ch)

Lead contact: S.M.-F. (sm2116@cam.ac.uk)

Figure S1

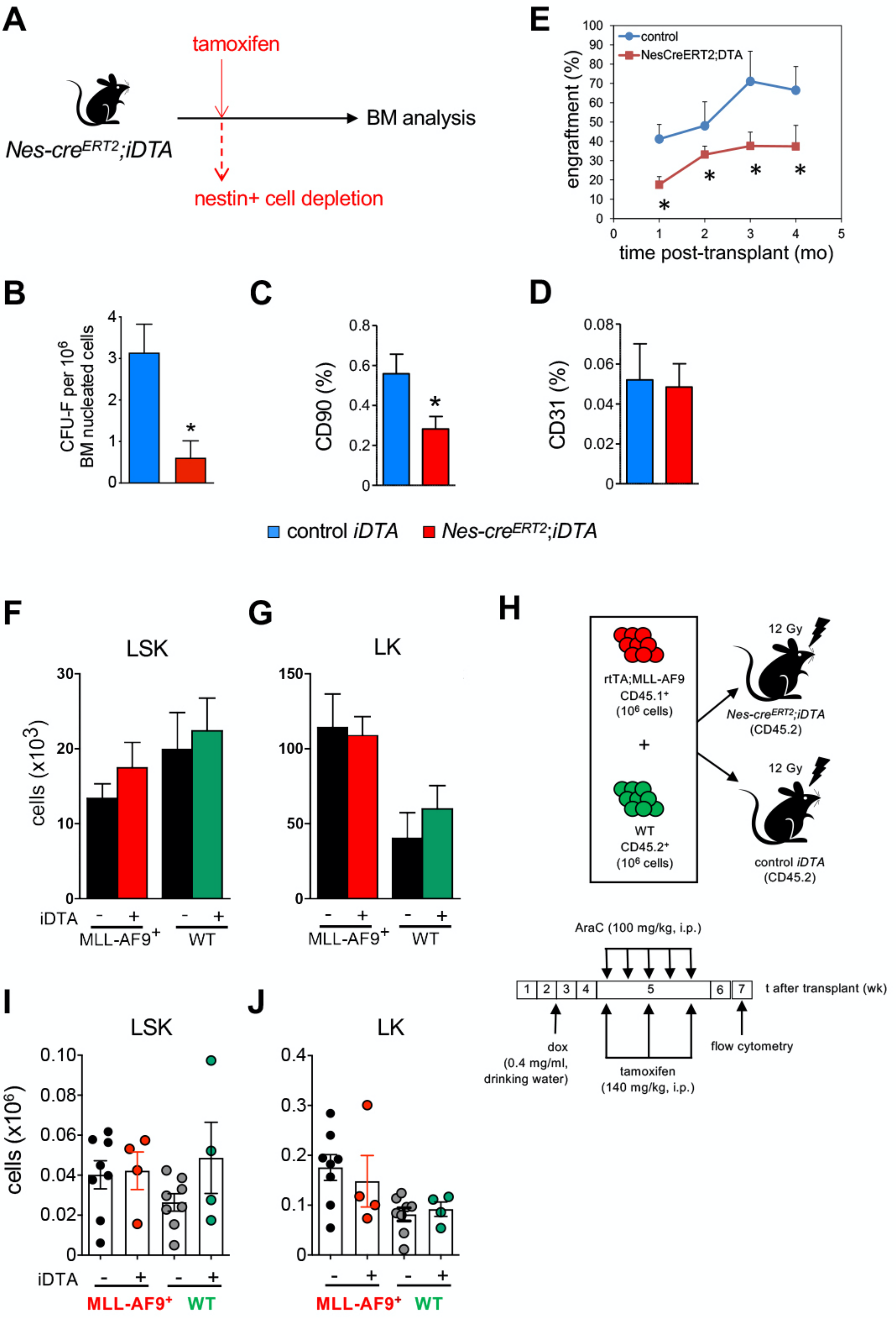
A
tamoxifen
Nes-creERT2;iDTA
BM analysis
nestin+ cell depletion
E
control
NesCreERT2;DTA
engraftment (%)
time post-transplant (mo)
B
CFU-F per 10^6 BM nucleated cells
C
CD90 (%)
D
CD31 (%)
control iDTA
Nes-creERT2;iDTA
F
LSK
cells (x10^3)
iDTA
MLL-AF9+
WT
G
LK
H
rtTA;MLL-AF9
CD45.1+
(10^6 cells)
WT
CD45.2+
(10^6 cells)
12 Gy
Nes-creERT2;iDTA
(CD45.2)
control iDTA
(CD45.2)
AraC (100 mg/kg, i.p.)
t after transplant (wk)
dox
(0.4 mg/ml,
drinking water)
tamoxifen
(140 mg/kg, i.p.)
flow cytometry
I
LSK
cells (x10^6)
J
LK

Figure S1. Related to Figure 1 and Figure 2. Nestin^{+} Cells Support AML Chemoresistance *in vivo*.

(A) Scheme of experimental depletion of nestin^{+} cells *in vivo*. Diphtheria toxin (iDTA) is induced in nestin+ cells (*Nes-cre^{ERT2}*) with tamoxifen. (B-D) Tamoxifen-treated *Nes-cre^{ERT2};iDTA* mice exhibit 2-fold-reduced BMSCs measured by fibroblastic-colony-forming units in culture (CFU-F, B) or flow cytometry (C, CD90^{+}) but unchanged frequency of CD31^{+} BM endothelial cells (D). (E) Long-term competitive repopulation assay using BM cells from tamoxifen-treated *Nes-cre^{ERT2};iDTA* mice or control mice. Half-reduced nestin^{+} cells cause a similar 2-fold reduction in functionally defined HSCs (n=4-5). Data are mean+SEM; *p<0.05; unpaired 2-tailed *t* test. (F-G) BM lin^{-} c-kit^{+} sca-1^{+} (LSK) cells (F) or lin^{-} c-kit^{hi} sca-1^{-} (LK) cells (G) in the same cohort of mice shown in Figure 2B-E. Data represent the cellularity of 4 limbs, sternum and spine (n=18 mice/group, pooled from 3 independent experiments). (H) Schematic of nestin^{+} cell depletion experiment in combination with standard chemotherapy treatment. Lethally-irradiated *Nes-cre^{ERT2};iDTA* or control *iDTA* mice were transplanted with iMLL-AF9;CD45.1^{+} BM and WT CD45.2^{+} BM (10^{6} cells each). Doxycycline induction began 2 weeks post-transplant. Mice were simultaneously treated with tamoxifen (140 mg/kg, i.p., 3 doses on alternate days) and cytarabine (5 daily doses of 100 mg/kg), starting 2 weeks after MLL-AF9 induction. (I-J) Number of BM LSK cells (I) or LK cells (J) in the same cohort of mice shown in Figure 2F-I). Data are mean ± SEM. *p < 0.05; **p < 0.01; unpaired 2-tailed *t* test.

Figure S2

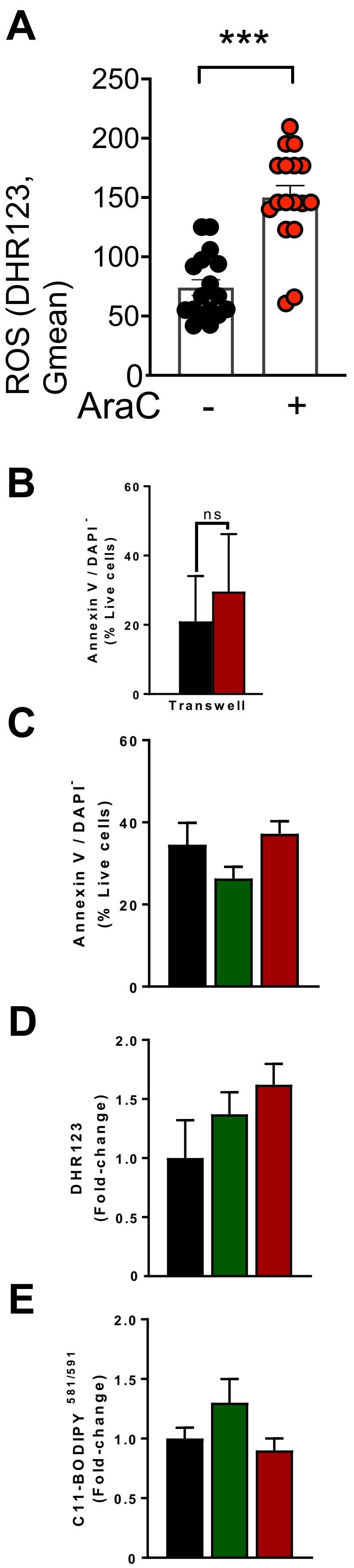

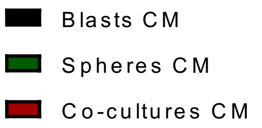

F

NAME	SIZE	ES	NES	NOM p-val	FDR q-val
[KEGG] RIBOSOME	80	0.660524	2.646718	0	0
[REACTOME] SRP DEPENDENT COTRANSLATIONAL PROTEIN TARGETING TO MEMBRANE	100	0.628575	2.639465	0	0
[REACTOME] PEPTIDE CHAIN ELONGATION	78	0.657575	2.639346	0	0
[REACTOME] 3 UTR MEDIATED TRANSLATIONAL REGULATION	96	0.614179	2.500665	0	0
[REACTOME] NONSENSE MEDIATED DECAY ENHANCED BY THE EXON JUNCTION COMPLEX	99	0.597617	2.462984	0	0
[REACTOME] TRANSLATION	135	0.547938	2.36469	0	0
BROWN_MYELOID CELL DEVELOPMENT UP	121	0.556386	2.352744	0	0
CHYLA_CBFA2T3 TARGETS DN	165	-0.59156	-2.66123	0	0
TAKAO_RESPONSE TO UVB RADIATION UP	78	0.571386	2.285674	0	0.000138
[REACTOME] METABOLISM OF MRNA	198	0.488466	2.253579	0	0.00021
BILANGES_SERUM AND RAPAMYCIN SENSITIVE GENES	64	0.577935	2.256664	0	0.000227
CHYLA_CBFA2T3 TARGETS UP	298	0.472523	2.269466	0	0.000248
[REACTOME] FORMATION OF THE TERNARY COMPLEX AND SUBSEQUENTLY THE 43S COMPLEX	42	0.642251	2.242198	0	0.000295
PECE_MAMMARY STEM CELL DN	99	0.537975	2.218671	0	0.000524
HUANG_GATA2 TARGETS UP	133	0.510723	2.222296	0	0.000559
[REACTOME] ANTIGEN PROCESSING CROSS PRESENTATION	63	0.573375	2.185608	0	0.000734
DIAZ_CHRONIC MYELOGENOUS LEUKEMIA DN	78	0.537912	2.165891	0	0.000824
KAMIKUBO_MYELOID CEBPA NETWORK	26	0.690026	2.170219	0	0.000864
[REACTOME] ACTIVATION OF THE MRNA UPON BINDING OF THE CAP BINDING COMPLEX AND EIFS...	50	0.585	2.163678	0	0.000911
WONG_MITOCHONDRIA GENE MODULE	204	0.461671	2.160487	0	0.000929
[REACTOME] RESPIRATORY ELECTRON TRANSPORT ATP SYNTHESIS BY CHEMIOSMOTIC COUPLING...	75	0.540714	2.144574	0	0.001392
[REACTOME] RESPIRATORY ELECTRON TRANSPORT	62	0.555249	2.134636	0	0.001603
LINDSTEDT_DENDRITIC CELL MATURATION D	45	0.589801	2.131768	0	0.001648
PID_TOLL ENDOGENOUS PATHWAY	22	0.686208	2.068535	0	0.004009
[REACTOME] TCA CYCLE AND RESPIRATORY ELECTRON TRANSPORT	108	0.482977	2.008723	0	0.007295
ZHAN_MULTIPLE MYELOMA DN	30	0.622787	2.010501	0	0.007427
[KEGG] OXIDATIVE PHOSPHORYLATION	96	0.489754	2.013311	0	0.007459
[BIOCARTA] PROTEASOME PATHWAY	28	0.607642	1.994204	0	0.009132
TAKEDA_TARGETS OF NUP98 HOXA9 FUSION 10D DN	68	0.51688	1.984435	0	0.01005
MOOTHA_VOXPHOS	81	0.487864	1.979709	0	0.010552
[REACTOME] ER PHAGOSOME PATHWAY	53	0.524368	1.977314	0	0.010672
COATES_MACROPHAGE M1 VS M2 DN	47	0.55315	1.948218	0	0.013486
MORI_PLASMA CELL UP	46	0.542614	1.945206	0.0019763	0.013976
DAZARD_RESPONSE TO UV NHEK UP	150	0.443037	1.936478	0	0.015607
VALK_AML CLUSTER 7	16	-0.75116	-2.07633	0	0.02163
DAVIES_MULTIPLE MYELOMA VS MGUS DN	23	0.627805	1.90994	0	0.022674
HADDAD_T LYMPHOCYTE AND NK PROGENITOR DN	39	0.54586	1.908156	0	0.02278
[REACTOME] METABOLISM OF RNA	242	0.407779	1.900496	0	0.024719
TAKEDA_TARGETS OF NUP98 HOXA9 FUSION 8D DN	107	0.446769	1.891038	0	0.027288
[REACTOME] AUTODEGRADATION OF THE E3 UBIQUITIN LIGASE COP1	46	0.522861	1.883418	0	0.029394
LIANG_HEMATOPOIESIS STEM CELL NUMBER LARGE VS TINY UP	37	0.544374	1.866835	0	0.032363
[REACTOME] CROSS PRESENTATION OF SOLUBLE EXOGENOUS ANTIGENS ENDOSOMES	43	0.52439	1.8722	0.0018904	0.032746
CHNG_MULTIPLE MYELOMA HYPERPLOID UP	50	0.513755	1.867433	0.0019493	0.033231
IVANOVA_HEMATOPOIESIS INTERMEDIATE PROGENITOR	129	0.434211	1.869	0	0.033279
TONKS_TARGETS OF RUNX1 RUNX1T1 FUSION HSC DN	142	0.424019	1.863782	0	0.033329
TARTE_PLASMA CELL VS B LYMPHOCYTE UP	67	0.483479	1.860026	0	0.034126
[REACTOME] CDK MEDIATED PHOSPHORYLATION AND REMOVAL OF CDC6	46	0.510727	1.855068	0.0018832	0.034147
[REACTOME] P53 INDEPENDENT G1 S DNA DAMAGE CHECKPOINT	48	0.508756	1.855126	0	0.034637
PARK_APL PATHOGENESIS DN	36	0.545674	1.857307	0	0.034833
ROSS_AML WITH CBFB MYH11 FUSION	35	0.552851	1.855453	0	0.034944
[REACTOME] ACTIVATION OF NF KAPPAB IN B CELLS	59	0.489852	1.850122	0	0.035877
RAMALHO_STEMNESS DN	58	0.484326	1.847525	0	0.03646
MUNSHI_MULTIPLE MYELOMA UP	64	0.484746	1.841195	0	0.038612
BASSO_CD40 SIGNALING UP	75	0.464023	1.835388	0	0.039448
DAZARD_UV RESPONSE CLUSTER G1	46	0.50898	1.836915	0	0.039805
[REACTOME] APC C CDH1 MEDIATED DEGRADATION OF CDC20 AND OTHER APC C CDH1 TARGETED PROTEINS..	64	0.472771	1.827783	0	0.040451
[KEGG] LEUKOCYTE TRANSENDOTHELIAL MIGRATION	74	0.460676	1.826143	0	0.04063
DUNNE_TARGETS OF AML1 MTG8 FUSION UP	39	0.527113	1.824601	0.0039216	0.040932
DAZARD_RESPONSE TO UV SCC UP	96	0.445608	1.828488	0	0.041237
PID_INTEGRIN2 PATHWAY	17	0.633346	1.818721	0.0018622	0.043433
ROSS_AML WITH MLL FUSIONS	57	0.479096	1.81594	0.0018975	0.044012
CHICAS_RB1 TARGETS LOW SERUM	60	0.477382	1.811473	0	0.044404
[REACTOME] APC C CDC20 MEDIATED DEGRADATION OF MITOTIC PROTEINS	65	0.470276	1.809885	0	0.044685
CREIGHTON_AKT1 SIGNALING VIA MTOR UP	28	0.562721	1.808257	0	0.044965
[REACTOME] AUTODEGRADATION OF CDH1 BY CDH1 APC C	56	0.47959	1.799929	0.0019305	0.048061
MARKEY_RB1 CHRONIC LOF DN	72	0.457876	1.797285	0	0.048201
[REACTOME] G ALPHA1213 SIGNALLING EVENTS	51	0.481115	1.793445	0.004008	0.048843

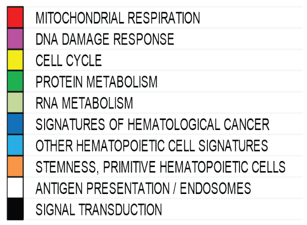

Figure S2. Related to Figure 3. Metabolic Profiling of AML Blasts and BMSCs Cultured Separately or Together Under AraC Treatment and Gene Sets Regulated by Nestin^{+} Cells in LK^{lo} AML Cells.

(A) Reactive oxygen species (ROS) measured by DH123 staining in AML blasts cultured alone in the absence/presence of AraC for 24h. Each dot is a biological replicate. ***p<0.01; unpaired two-tailed *t* test. (B-E) Transwell coculture or use of conditioned medium from mesenspheres cannot protect AML blasts for AraC-induced cell death, excessive ROS or lipid peroxidation. (B) Frequency of alive AML blasts in monoculture (black column) or transwell cocultures (red) with BM mesenshperes under AraC treatment. (C-E) Conditioned medium (CM) from mesenspheres or cocultures cannot protect AML blasts from AraC-induced cell death (C), excessive ROS levels (D) or lipid peroxidation (E). Leukemic blasts in monoculture (black column) (200 x 10^3 cells/ml) and mouse nestin+ mesenspheres (~250/ml) were cultured alone or in co-culture for 24 h. CM was collected to seed new leukemic blasts (with CM from previous cultures) for 24h. Detection of apoptosis with Annexin V/DAPI (C), intracellular ROS production with DHR123 expressed as fold-change to control with CM from leukemic blasts (D), lipid peroxidation using C(11)-BODIPY(581/591) (E) were measured by flow cytometry in the leukemic blasts. One-way ANOVA followed by post-hoc multiple comparison. Data are mean ± SEM. (F) Gene Sets Regulated by Nestin^{+} Cells in LK^{lo} AML Cells. Output of a gene-set enrichment analysis (GSEA) of gene expression profiling data from LK^{lo} cells sorted from control or *Nes-cre^{ERT2};iDTA* mice transplanted with WT and iMLL-AF9 BM cells. The table represents those gene sets most significantly enriched (FDR < 0.05) in one of the genotypes. Positive enrichment scores indicate enrichment in control cells; negative scores mean enrichment in cells from *Nes-cre^{ERT2};iDTA* mice. Gene sets were color-coded according to the cellular process to which they are most related. A number of gene sets of little relevance (mainly signatures of various non-hematological diseases including solid tumors) were omitted for clarity. ES, enrichment score; NES, normalized enrichment score; NOM p-val, nominal (unadjusted) p-value; FDR, Benjamini-Hochberg false discovery rate.

Figure S3

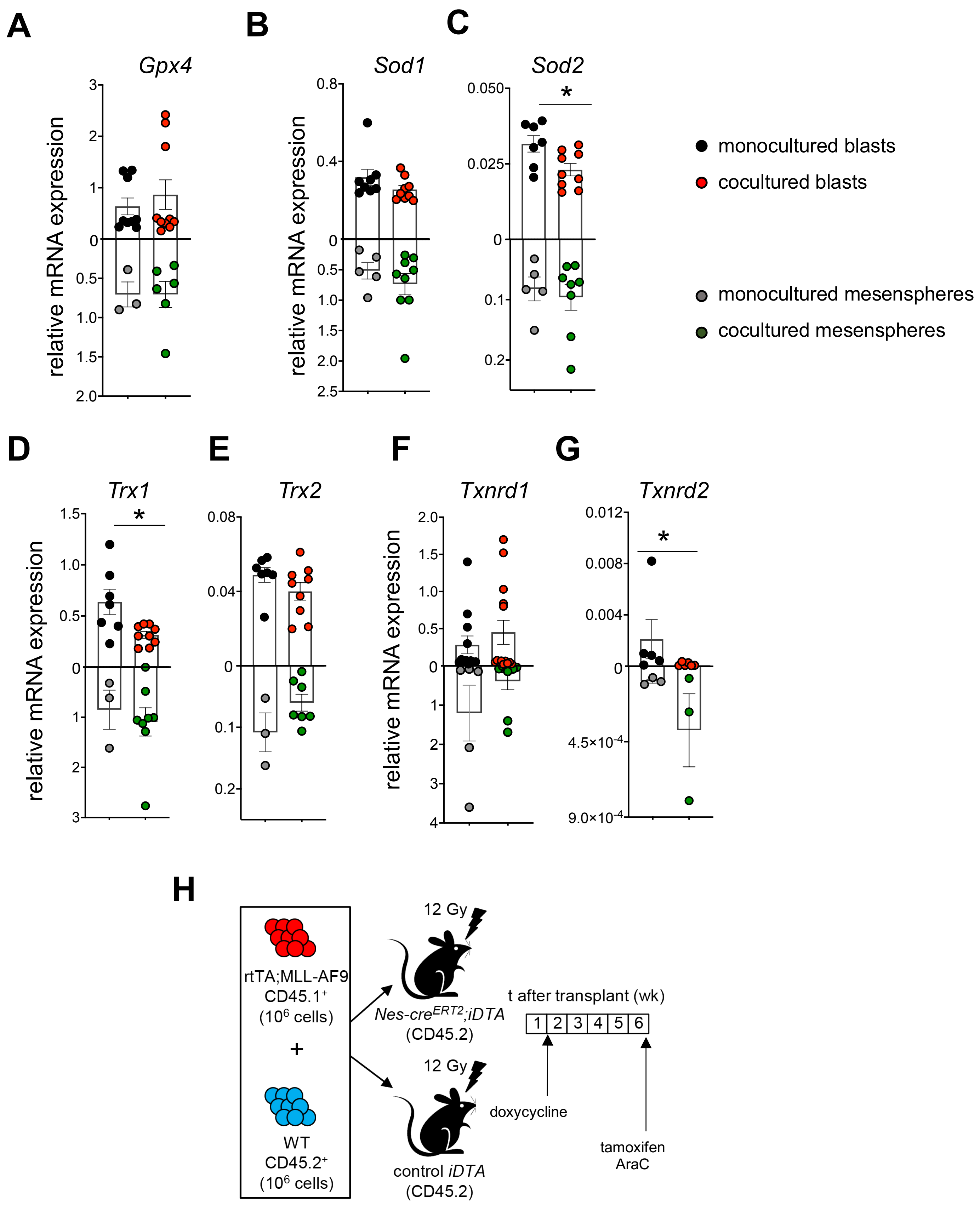
A
B
C
D
E
F
G
H
Gpx4
Sod1
Sod2
Trx1
Trx2
Txnrd1
Txnrd2
relative mRNA expression
*
monocultured blasts
cocultured blasts
monocultured mesenspheres
cocultured mesenspheres
rtTA;MLL-AF9
CD45.1+
(10^6 cells)
+
WT
CD45.2+
(10^6 cells)
12 Gy
Nes-creERT2;iDTA
(CD45.2)
control iDTA
(CD45.2)
t after transplant (wk)
1 2 3 4 5 6
doxycycline
tamoxifen
AraC

Figure S3. Related to Figure 5. mRNA Expression of Genes Related to Antioxidant Defense in AML Blasts and BMSCs.

mRNA expression of genes encoding (A) glutathione peroxidase 4 (*Gpx4*) , superoxide dismutases 1 (*Sod1*, B) and 2 (*Sod2*, C), thioxiredoxins 1 (*Trx1*, D) and 2 (*Trx2*, E), and thioredoxin reductases 1 (*Txnrd1*, F) and 2 (*Txnrd1*, G) in AML blasts and BMSCs cultured alone or together for 24h in presence of AraC. (H) Scheme showing the *in vivo* experimental paradigm to study the impact of nestin$^+$ cell depletion on GSH-dependent antioxidant AML protection from AraC.

Figure S4

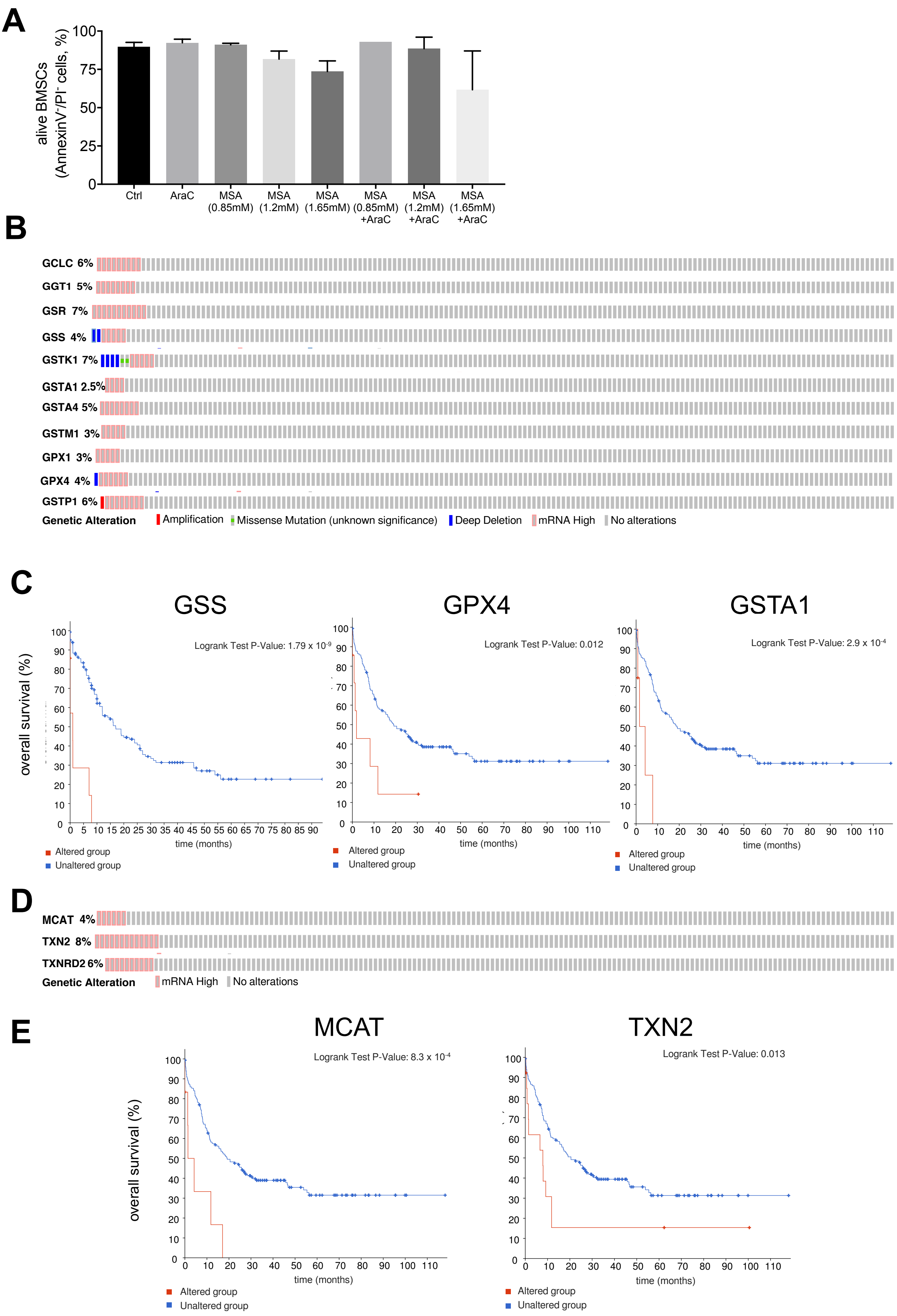
A
alive BMSCs (AnnexinV-/PI- cells, %)
Ctrl
AraC
MSA (0.85mM)
MSA (1.2mM)
MSA (1.65mM)
MSA (0.85mM) +AraC
MSA (1.2mM) +AraC
MSA (1.65mM) +AraC
B
GCLC 6%
GGT1 5%
GSR 7%
GSS 4%
GSTK1 7%
GSTA1 2.5%
GSTA4 5%
GSTM1 3%
GPX1 3%
GPX4 4%
GSTP1 6%
Genetic Alteration
Amplification
Missense Mutation (unknown significance)
Deep Deletion
mRNA High
No alterations
C
GSS
GPX4
GSTA1
overall survival (%)
Logrank Test P-Value: 1.79 x 10-9
Logrank Test P-Value: 0.012
Logrank Test P-Value: 2.9 x 10-4
time (months)
Altered group
Unaltered group
D
MCAT 4%
TXN2 8%
TXNRD2 6%
Genetic Alteration
mRNA High
No alterations
E
MCAT
TXN2
overall survival (%)
Logrank Test P-Value: 8.3 x 10-4
Logrank Test P-Value: 0.013
time (months)
Altered group
Unaltered group

Figure S4. Related to Figure 6. Increased mRNA Expression of Antioxidant-Related Genes Correlates with Poor Overall Survival in Human AML.

(A) Alive BMSCs after treatment with AraC and/or various concentrations of mercaptosuccinic acid (MSA) for 24h. (B, D) List of genes involved in antioxidant pathways and frequency of AML patients with abnormalities in these genes (Cancer Genome Atlas Research, 2013). (C, E) Correlation with overall survival from public database TCGA. List of interrogated genes included (B) GSH-related molecules: GCLC, catalytic subunit of glutamate-cysteine ligase; GGT1, Gamma-glutamyltransferase 1; GSR, glutathione reductase; GSS, glutathione oxidase; GSTK1, Glutathione S-transferase kappa 1; GSTA1, Glutathione S-transferase alpha 1; GSTA4, Glutathione S-transferase alpha 4; GSTM1, Glutathione S-transferase Mu 1; GPX1, glutathione peroxidase 1; GPX4, glutathione peroxidase 4; GSTP1, Glutathione S-transferase P; (E) Other antioxidant-related molecules: MCAT, Malonyl-CoA-Acyl carrier protein transacylase; TXN2, thioredoxin 2; TXNRD2, thioredoxin reductase 2. Genetic alterations include amplification (red box), missense mutation (green box), deep deletion (blue box) or mRNA upregulation (empty red box). Increased mRNA expression of (C) GSS, GPX4, GSTA1, (E) MCAT or TXN2 correlates with poor overall survival in human AML.

Table S1. Related to Figure 4. Analysis of the RNAseq data from Nestin$^+$ BMSCs obtained from leukemic iMLL-AF9 and normal mice. BM stromal (CD45$^-$ Ter119$^-$CD31$^-$) Nes-GFP$^+$ cells were sorted from control normal (N) *Nes*-GFP*;rtTA* mice and leukemic (L) *Nes*-GFP*;rtTA;iMLL-AF9* mice. N2, N3 correspond to normal mice; L2, L3 correspond to leukemic mice. Each sample comprised cells obtained from one or two mice. The first two sheets correspond to genes significantly up- or down-regulated in nestin$^+$ BMSCs from leukemic mice. The other sheets list pathways analysis taking into consideration differentially expressed genes. Significant pathways for each subontology are shown in separated sheets (BP, biological process; MF, molecular function; CC, cellular component).

Table S2. Related to Figure 4. Analysis of the RNAseq data from leukemic BM lin$^-$ ckit^{lo} cells obtained from mice with/without nestin$^+$ cell depletion. Lethally-irradiated CD45.2 control mice or *Nes-cre^{ERT2};iDTA* mice were transplanted with 10^6 iAML (*rtTA;MLL-AF9*) CD45.2$^+$ BM nucleated cells and 10^6 CD45.1$^+$ WT BM nucleated cells. Doxycycline administration started 2 weeks after transplant; tamoxifen was administered 4 weeks post-transplant and mice were sacrificed and analyzed 4 weeks later. BM MLL-AF9$^+$ lin$^-$ ckit^{low} (LK^{lo}) cells were sorted from leukemic mice with nestin$^+$ cell depletion (E, experimental) or without nestin$^+$ cell depletion (C, control). The list of genes, their fragments per kilobase of exon model per million reads mapped (FKPM) values for individual control or experimental mice, the average values, fold change, p value and adjusted p values are indicated.

Table S3. Related to Figure 4. Analysis of the proteomic data from leukemic blasts and spheres in monoculture or coculture upon AraC treatment. AML blasts were cultured alone or cocultured with mesenspheres for 24h in the presence of AraC. The mesenspheres were mechanically isolated through filtration and the leukemic blast were sorted as CD45$^+$ DAPI$^-$ cells. The samples were processed for Quantitative Protein Analysis as described in the Methods. The sheet contains a list of proteins significantly up- or down-regulated in blasts or spheres and categories significantly regulated according to pathway enrichment analysis.
